# Supplementary figures and images for: Phosphatase PTPN22 functions as an adaptor in the mTORC2 complex
Source: EMBO Rep. 2025 Sep 16;26(21):5172–98. doi: 10.1038/s44319-025-00576-5 (PMC12592532; doi:10.1038/s44319-025-00576-5)

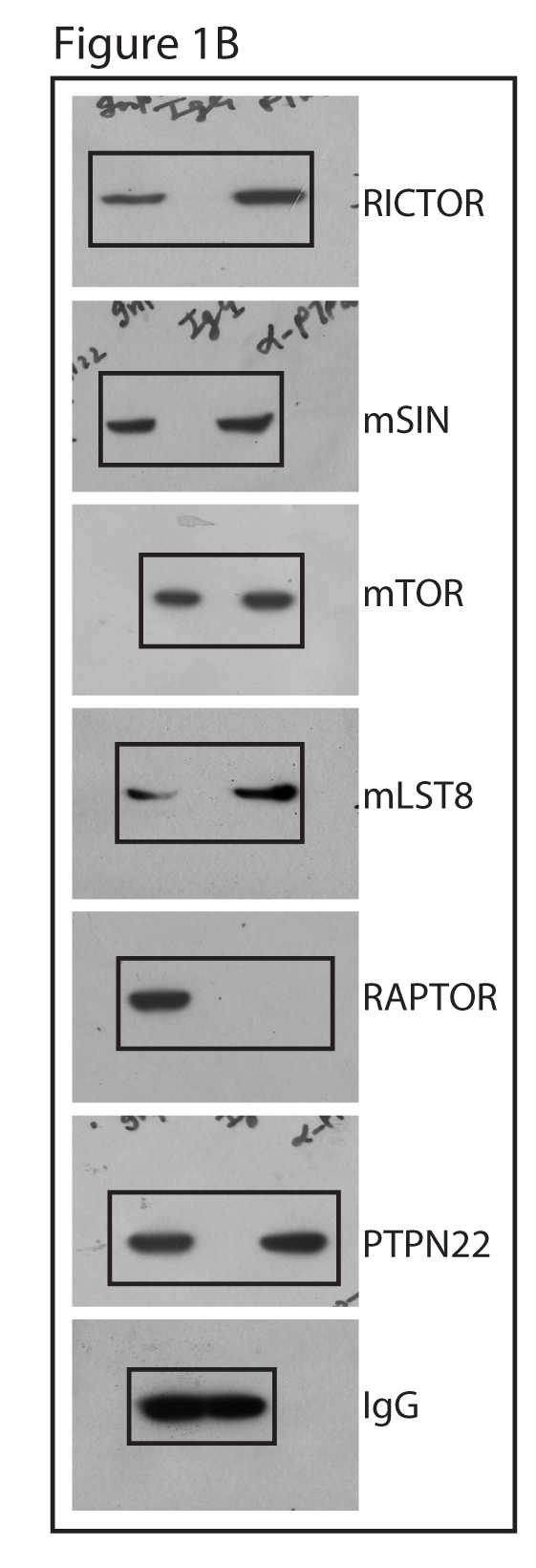

Supplement: Supplementary file 2 — Source data Fig. 1 [file 44319_2025_576_MOESM2_ESM.zip › Figure 1/1B/1B.tif]

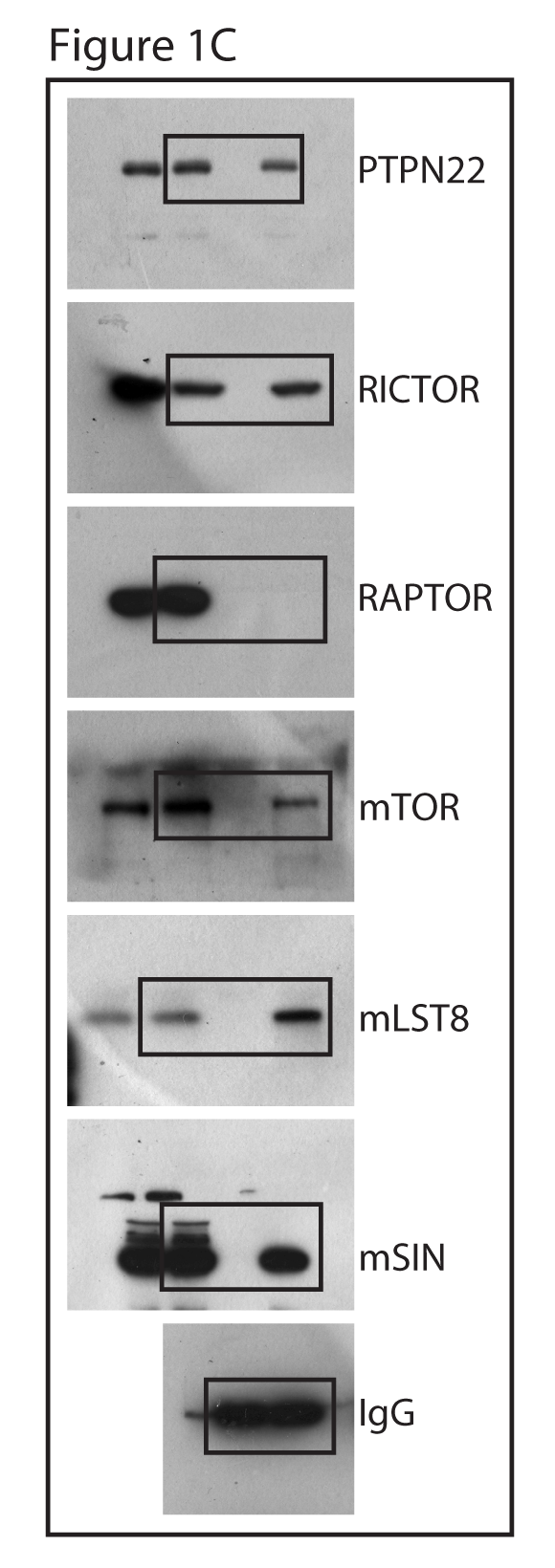

Supplement: Supplementary file 2 — Source data Fig. 1 [file 44319_2025_576_MOESM2_ESM.zip › Figure 1/1C/1C.tif]

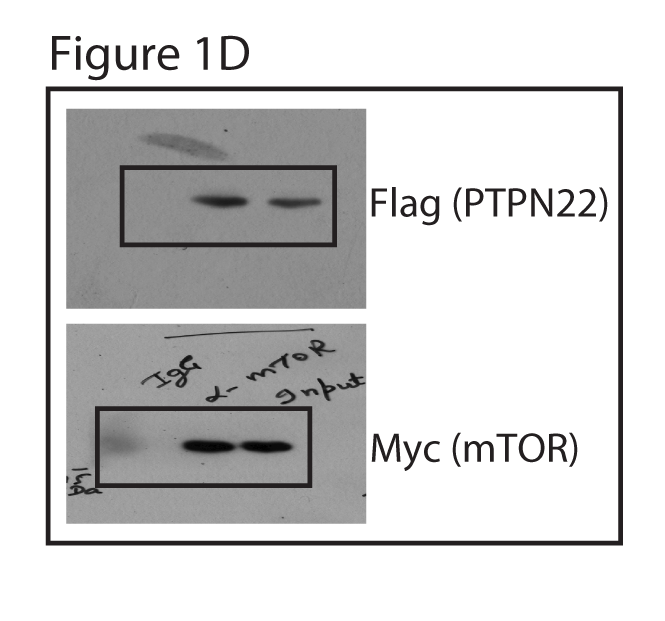

Supplement: Supplementary file 2 — Source data Fig. 1 [file 44319_2025_576_MOESM2_ESM.zip › Figure 1/1D/1D.tif]

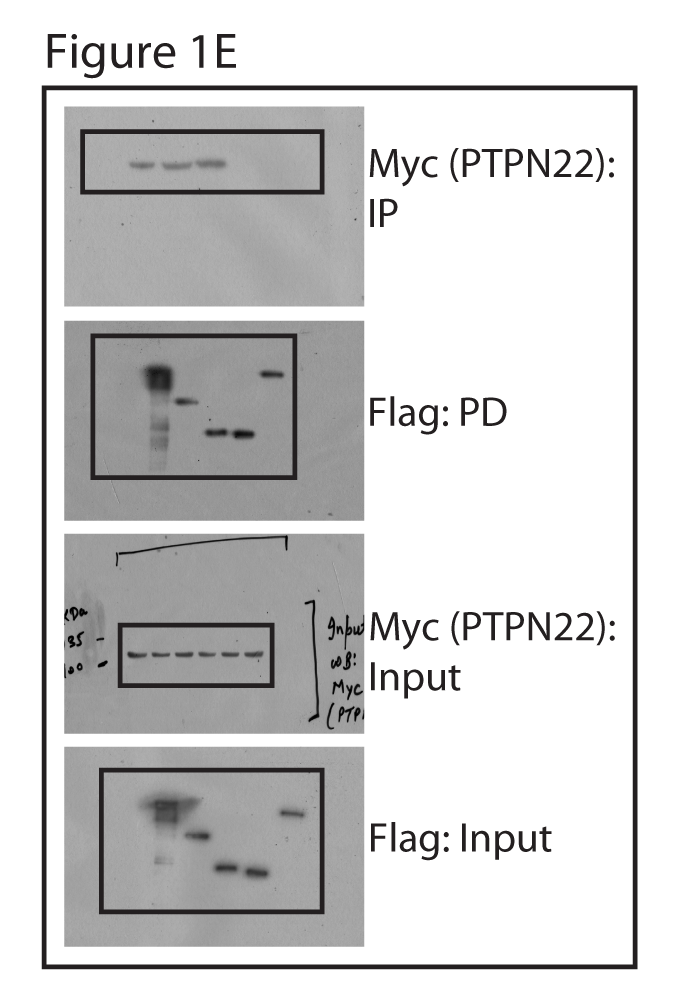

Supplement: Supplementary file 2 — Source data Fig. 1 [file 44319_2025_576_MOESM2_ESM.zip › Figure 1/1E/1E.tif]

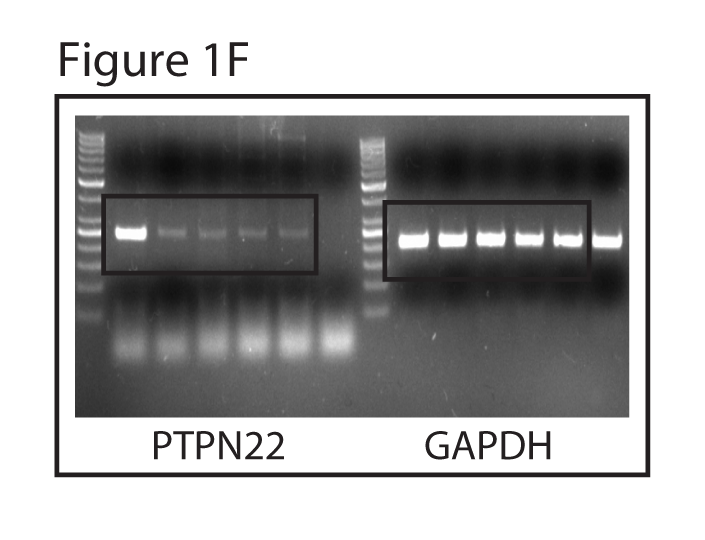

Supplement: Supplementary file 2 — Source data Fig. 1 [file 44319_2025_576_MOESM2_ESM.zip › Figure 1/1F/1F.tif]

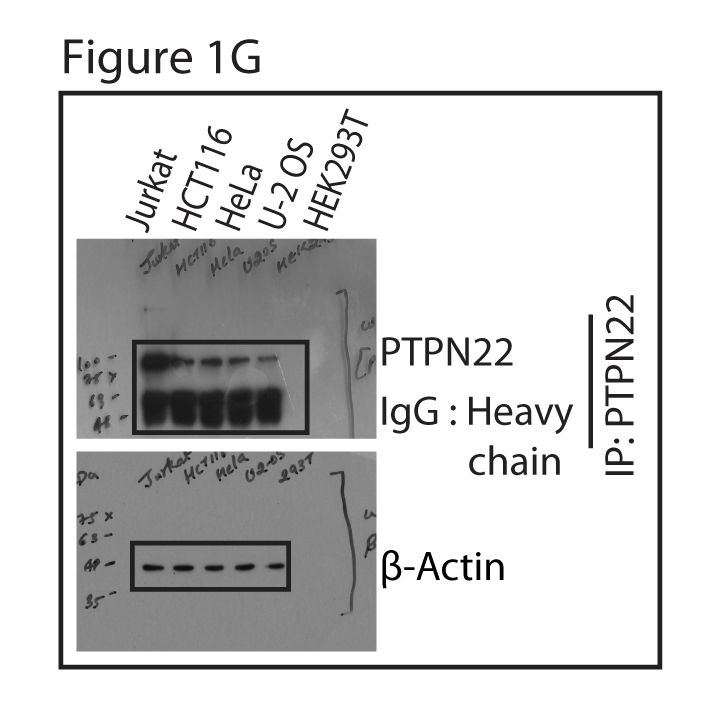

Supplement: Supplementary file 2 — Source data Fig. 1 [file 44319_2025_576_MOESM2_ESM.zip › Figure 1/1G/1G.tif]

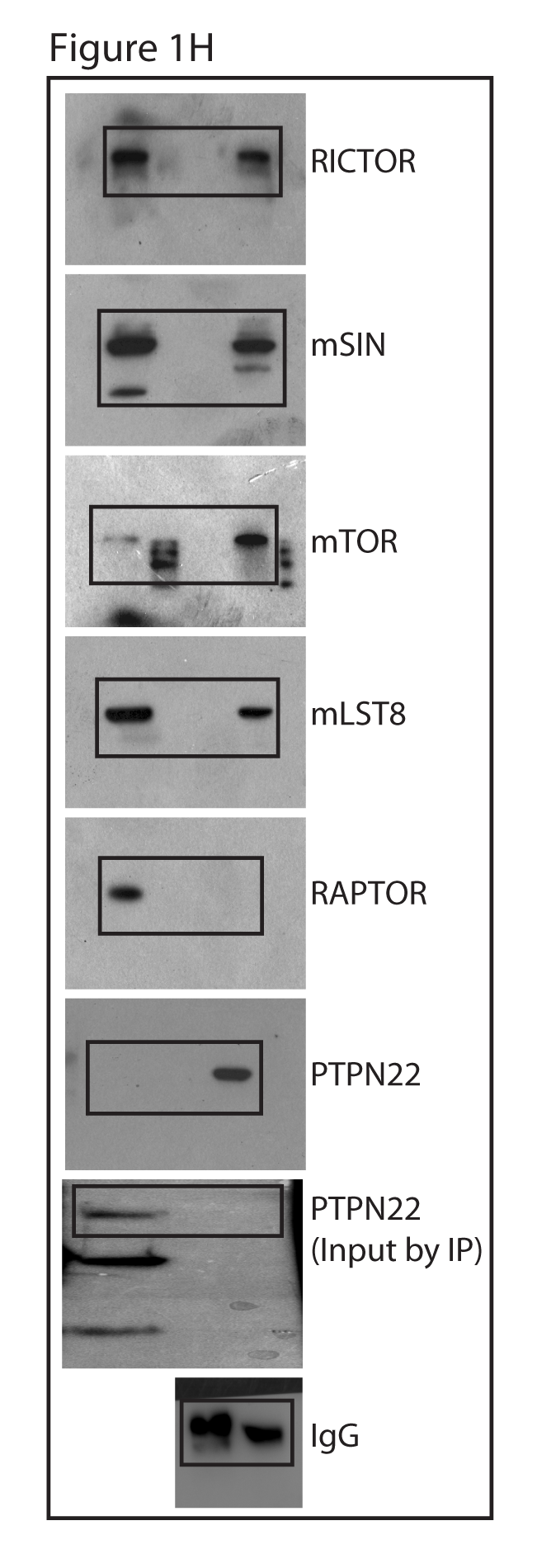

Supplement: Supplementary file 2 — Source data Fig. 1 [file 44319_2025_576_MOESM2_ESM.zip › Figure 1/1H/1H.tif]

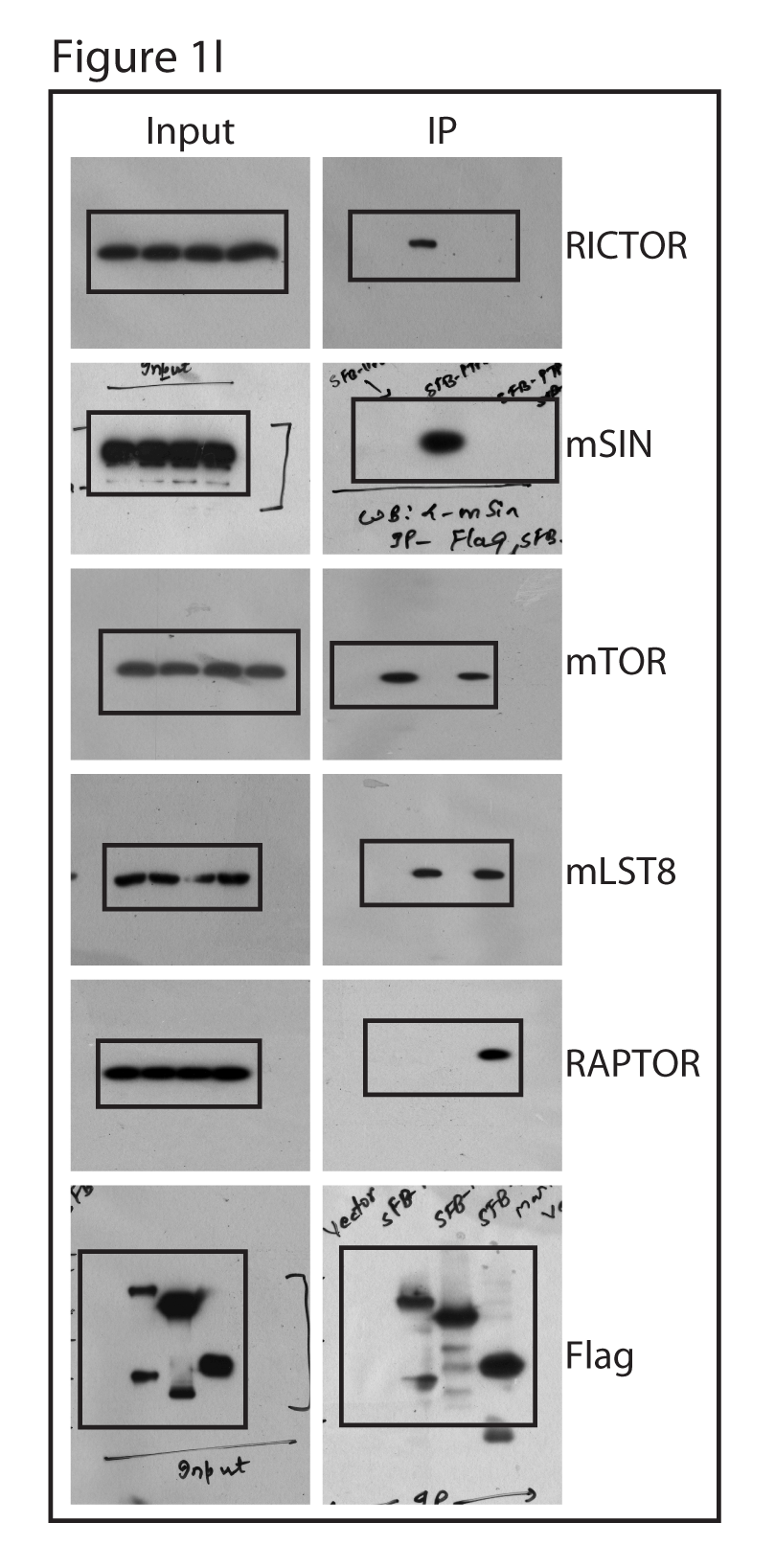

Supplement: Supplementary file 2 — Source data Fig. 1 [file 44319_2025_576_MOESM2_ESM.zip › Figure 1/1I/1I.tif]

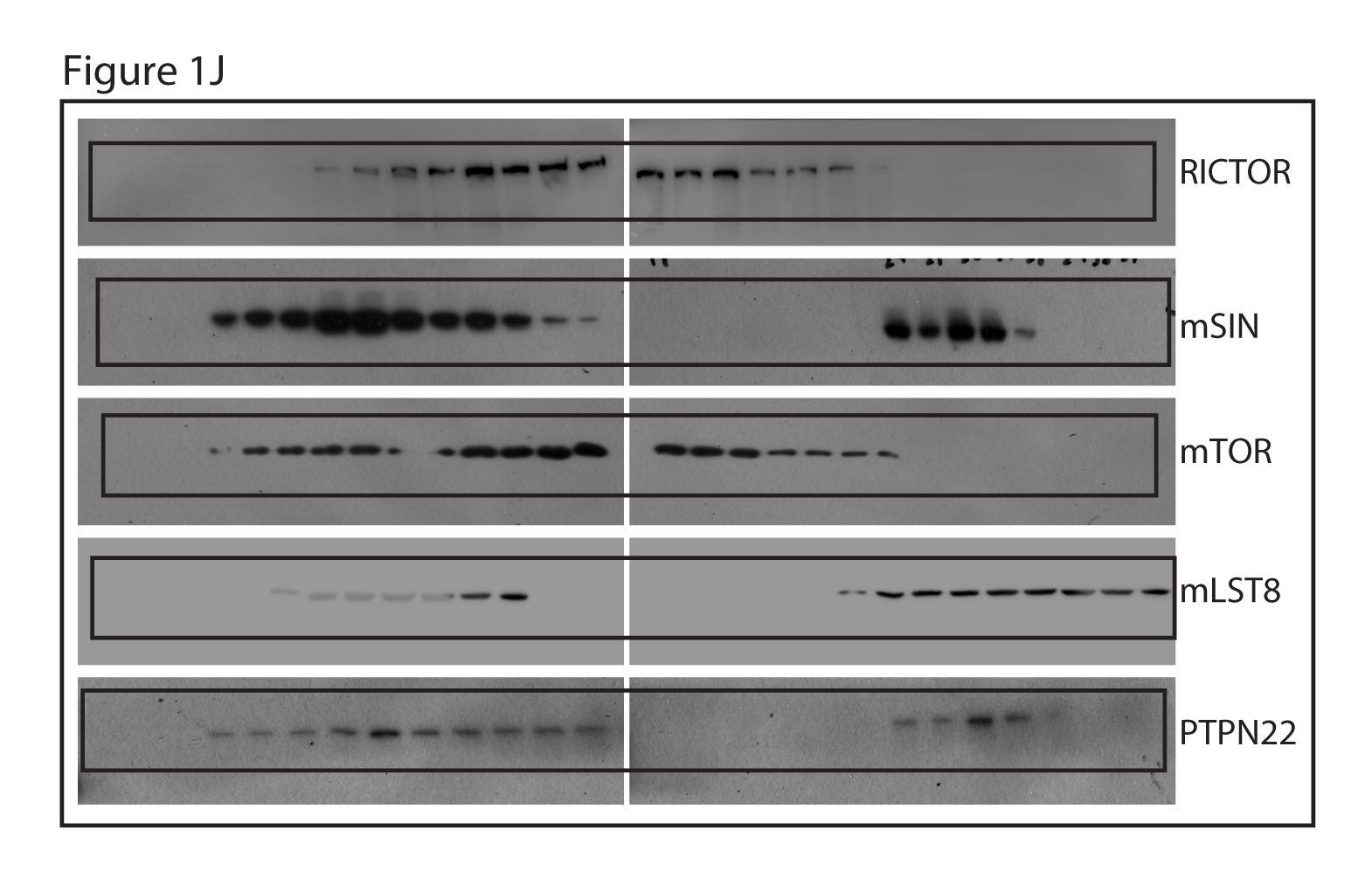

Supplement: Supplementary file 2 — Source data Fig. 1 [file 44319_2025_576_MOESM2_ESM.zip › Figure 1/1J/1J.tif]

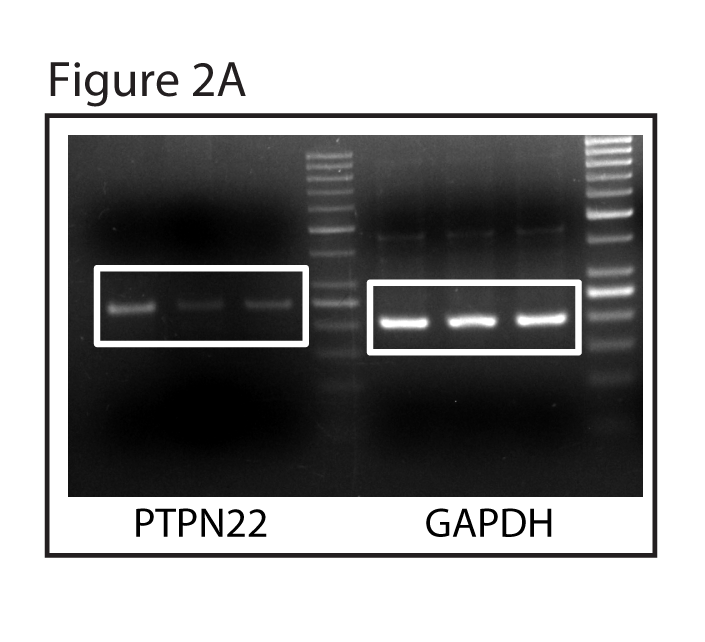

Supplement: Supplementary file 3 — Source data Fig. 2 [file 44319_2025_576_MOESM3_ESM.zip › Figure 2/2A/2A.tif]

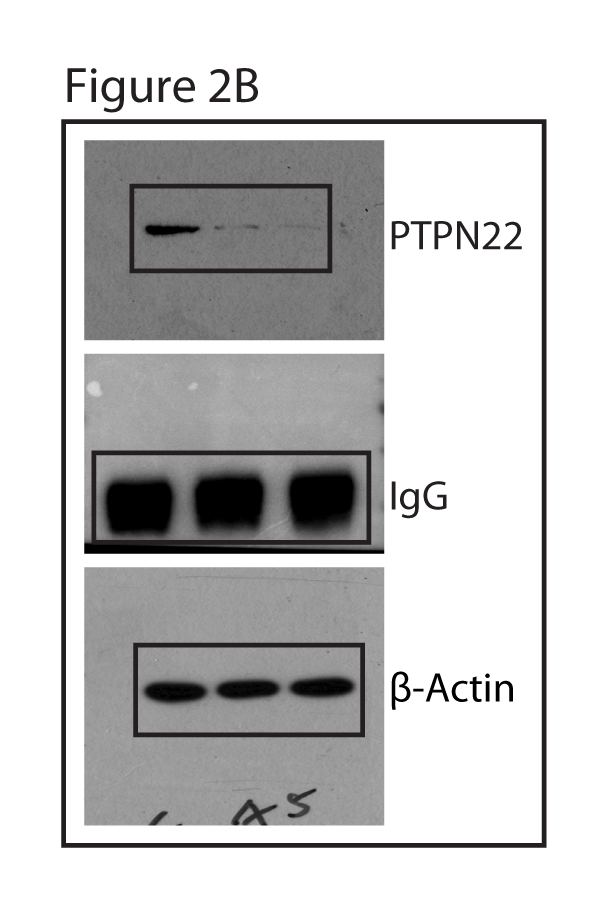

Supplement: Supplementary file 3 — Source data Fig. 2 [file 44319_2025_576_MOESM3_ESM.zip › Figure 2/2B/2B.tif]

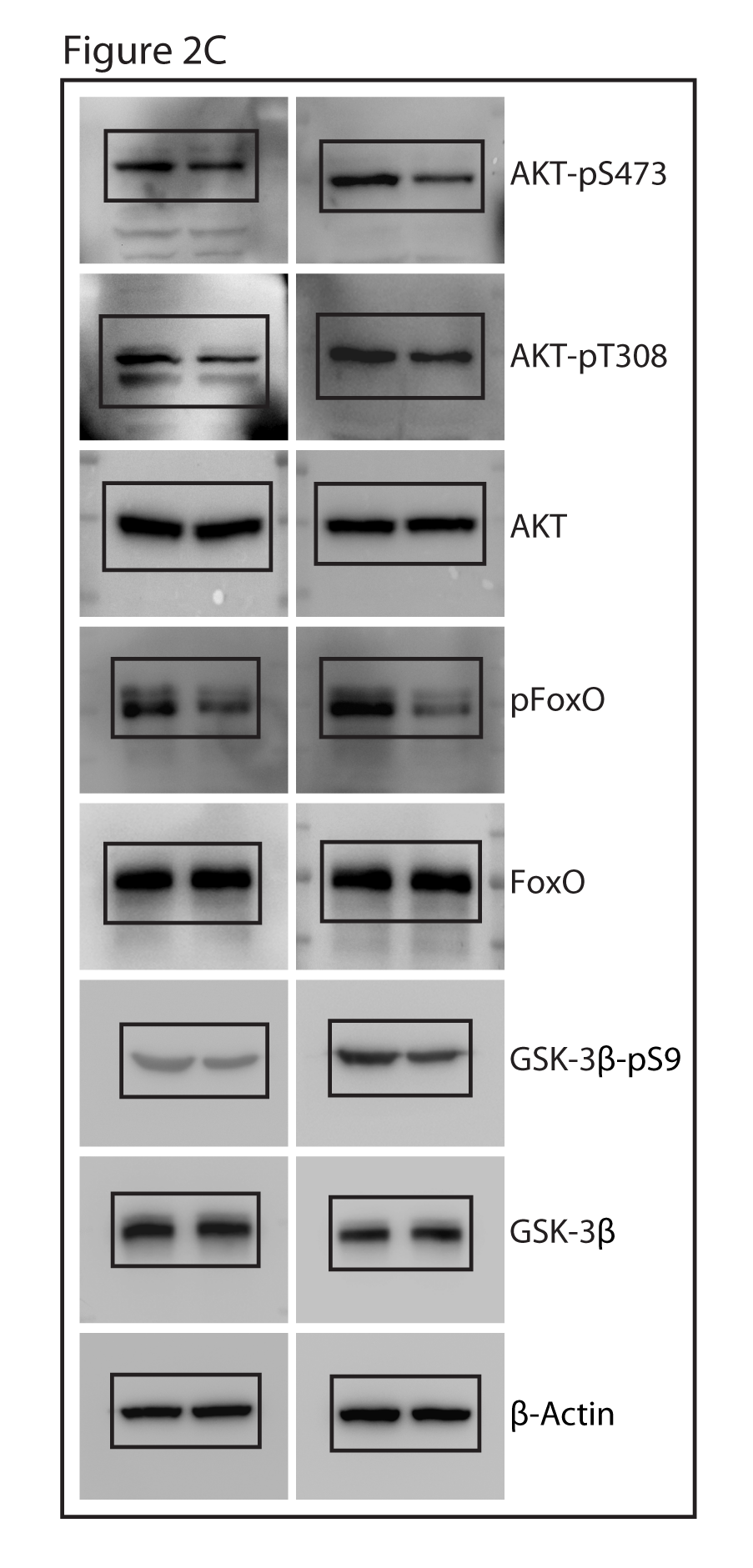

Supplement: Supplementary file 3 — Source data Fig. 2 [file 44319_2025_576_MOESM3_ESM.zip › Figure 2/2C/2C_Western Blots.tif]

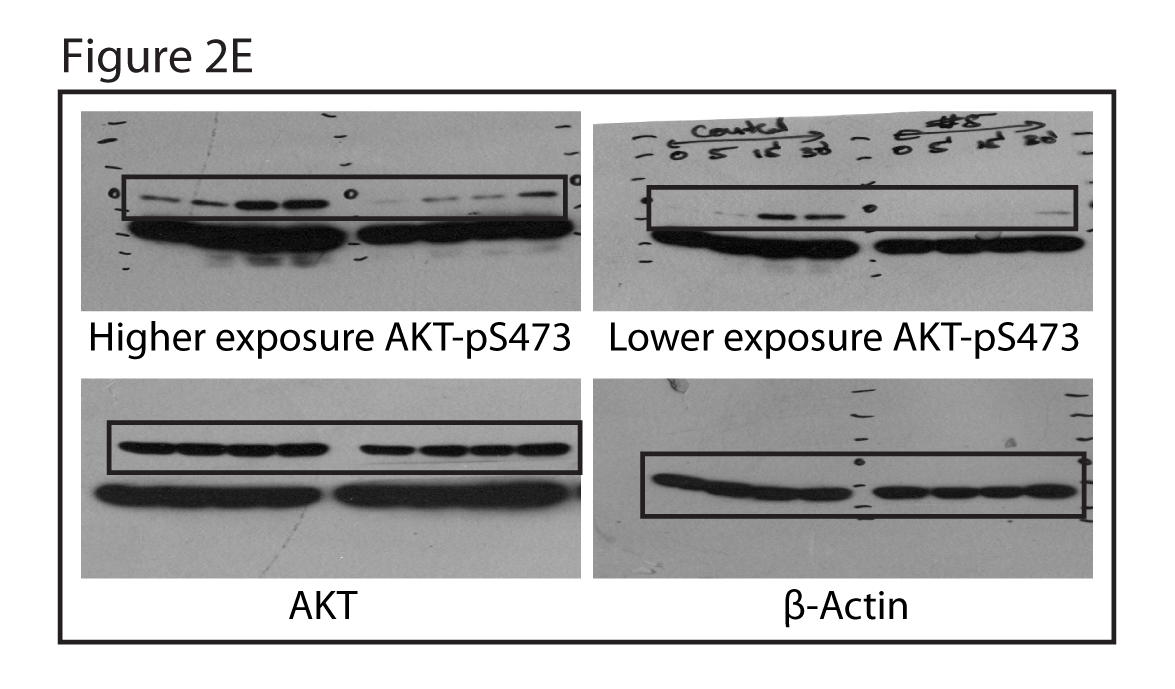

Supplement: Supplementary file 3 — Source data Fig. 2 [file 44319_2025_576_MOESM3_ESM.zip › Figure 2/2E/2E.tif]

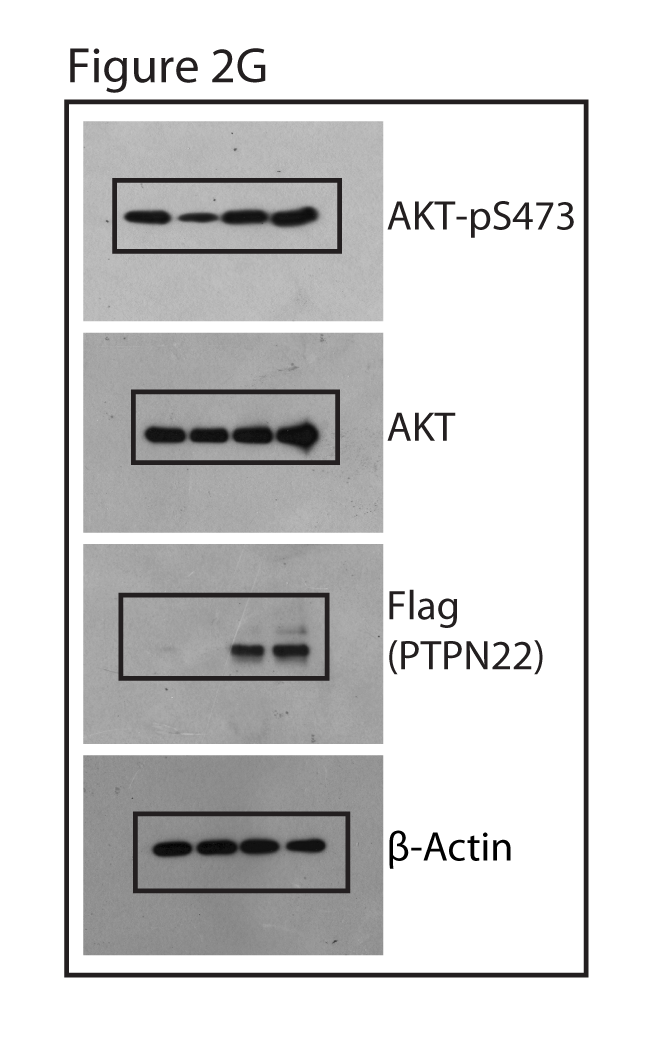

Supplement: Supplementary file 3 — Source data Fig. 2 [file 44319_2025_576_MOESM3_ESM.zip › Figure 2/2G/2G.tif]

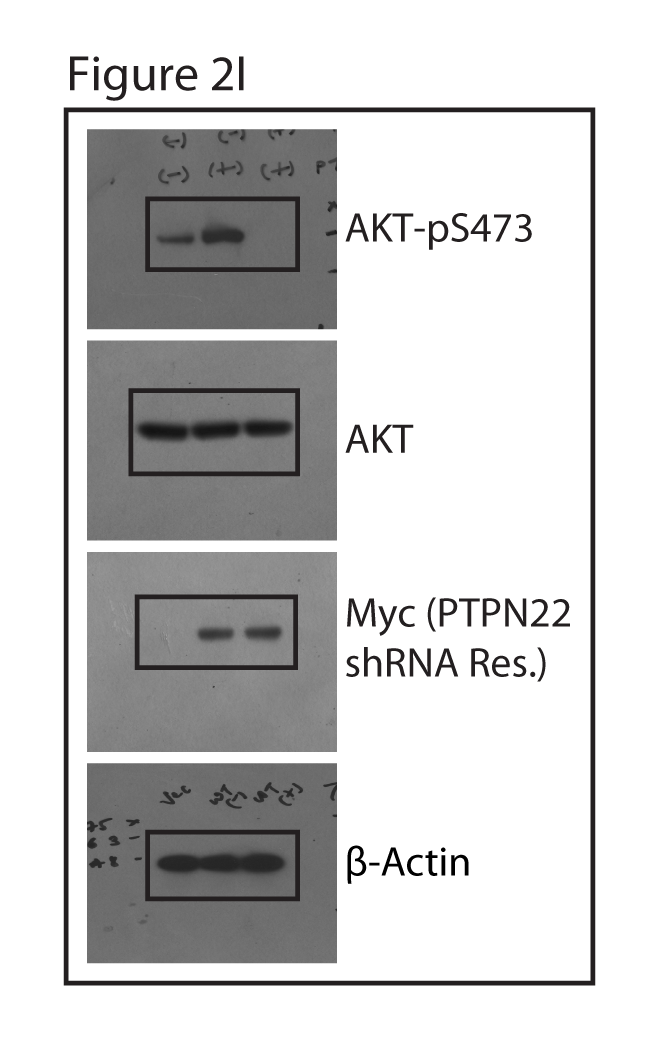

Supplement: Supplementary file 3 — Source data Fig. 2 [file 44319_2025_576_MOESM3_ESM.zip › Figure 2/2I/2I.tif]

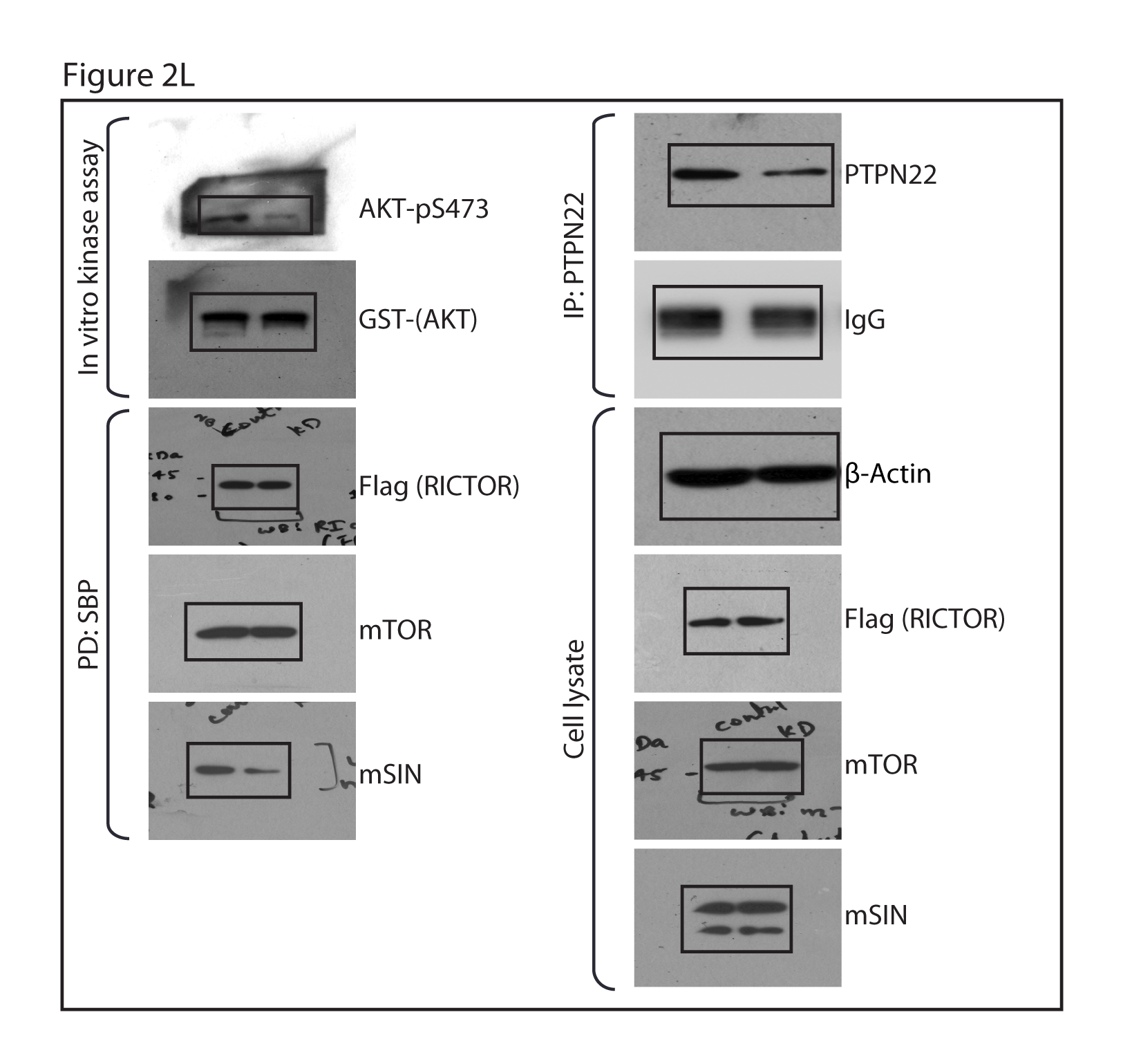

Supplement: Supplementary file 3 — Source data Fig. 2 [file 44319_2025_576_MOESM3_ESM.zip › Figure 2/2L/2L.tif]

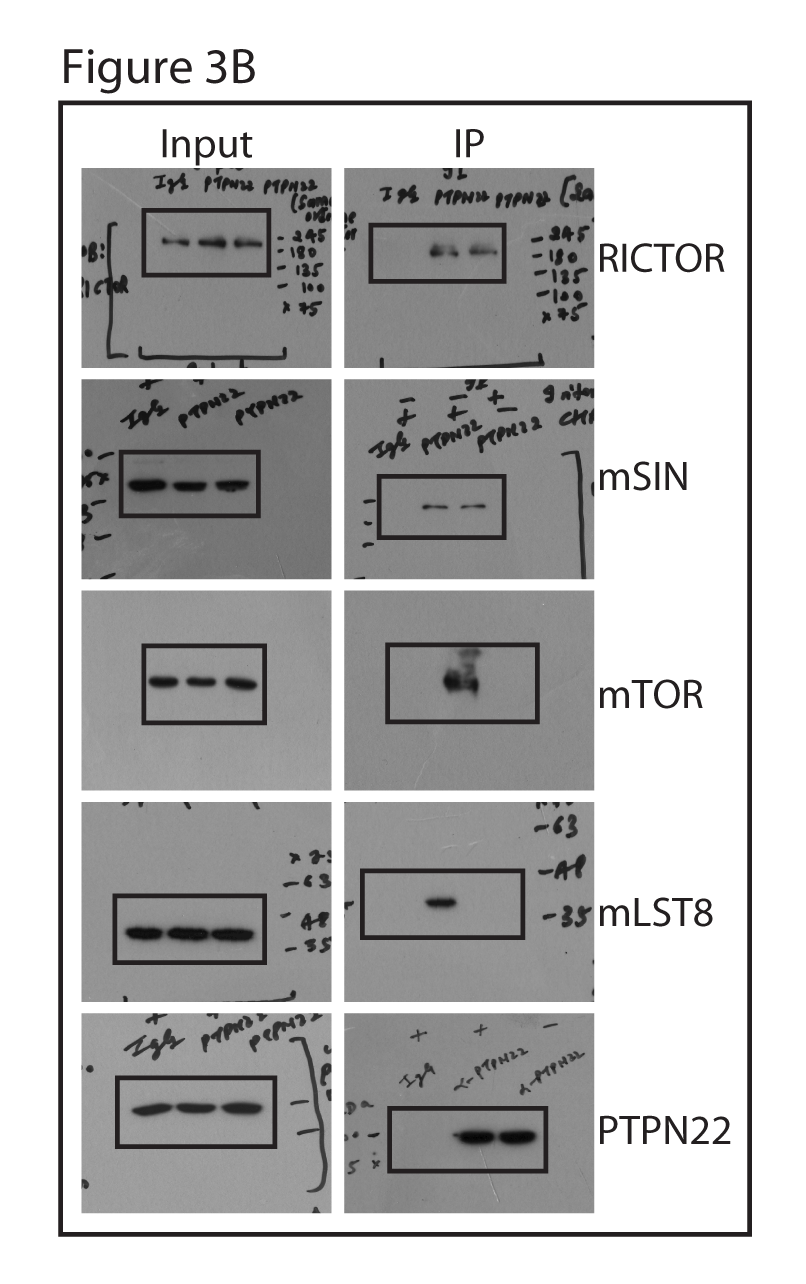

Supplement: Supplementary file 4 — Source data Fig. 3 [file 44319_2025_576_MOESM4_ESM.zip › Figure 3/3B/3B.tif]

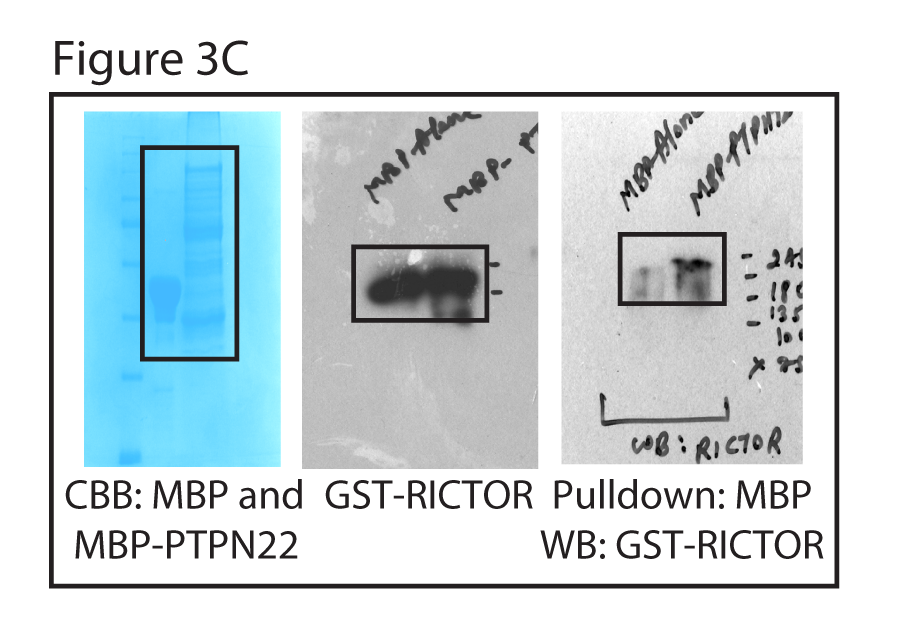

Supplement: Supplementary file 4 — Source data Fig. 3 [file 44319_2025_576_MOESM4_ESM.zip › Figure 3/3C/3C.tif]

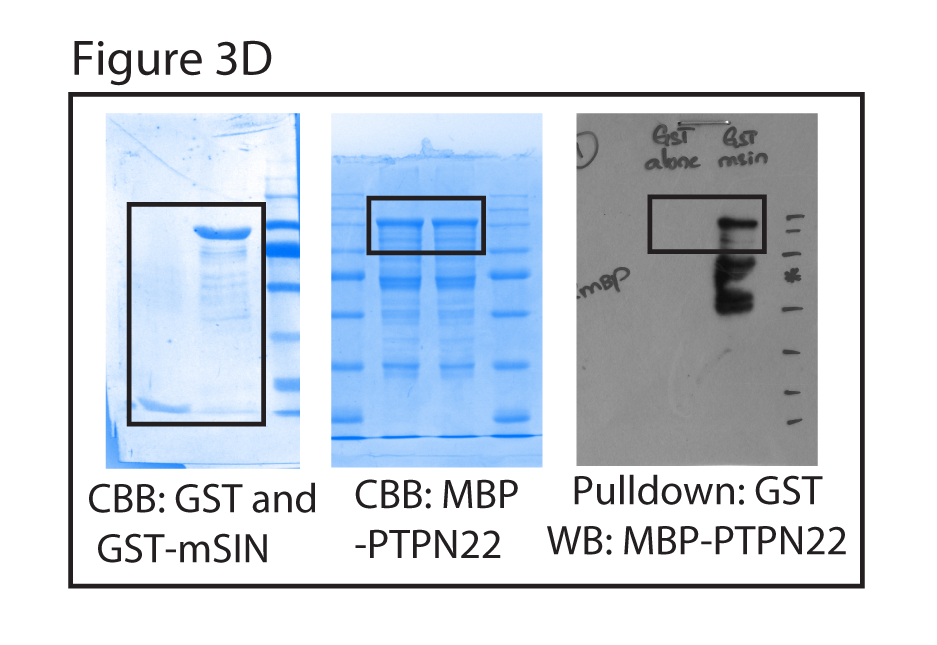

Supplement: Supplementary file 4 — Source data Fig. 3 [file 44319_2025_576_MOESM4_ESM.zip › Figure 3/3D/3D.tif]

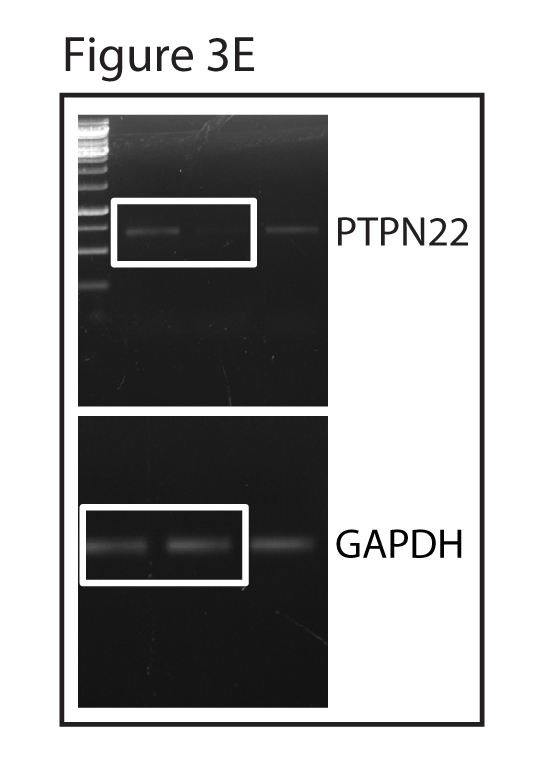

Supplement: Supplementary file 4 — Source data Fig. 3 [file 44319_2025_576_MOESM4_ESM.zip › Figure 3/3E/3E.tif]

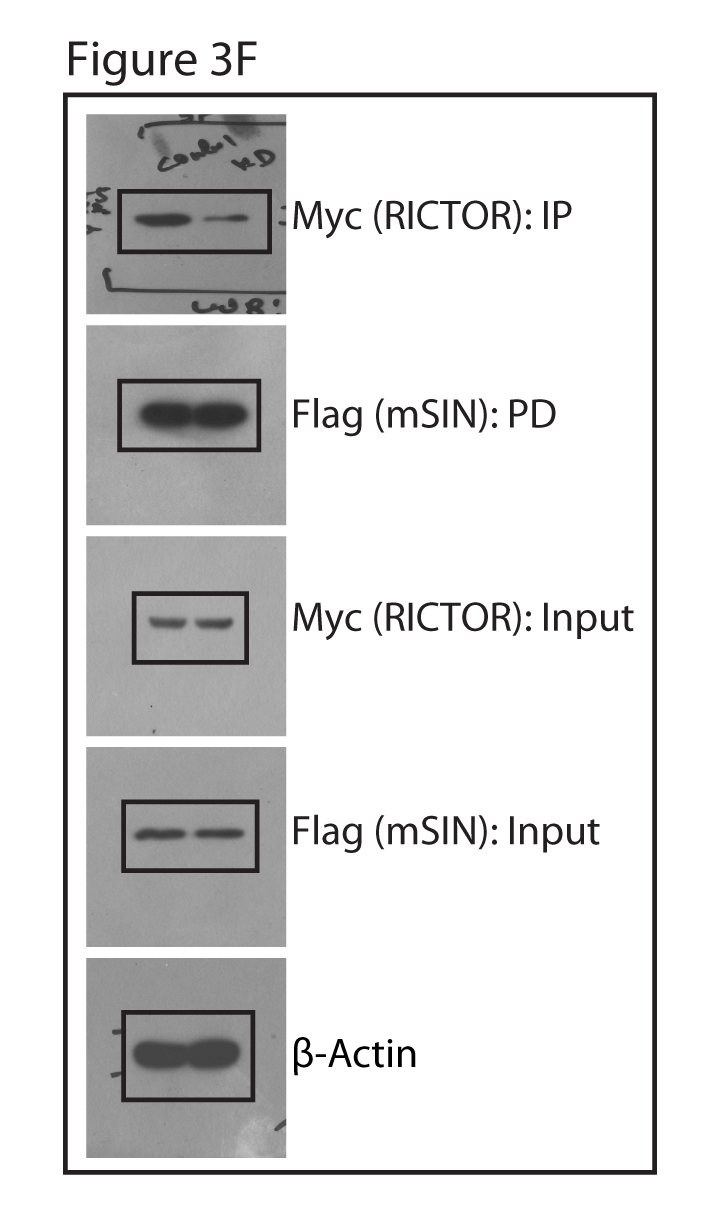

Supplement: Supplementary file 4 — Source data Fig. 3 [file 44319_2025_576_MOESM4_ESM.zip › Figure 3/3F/3F.tif]

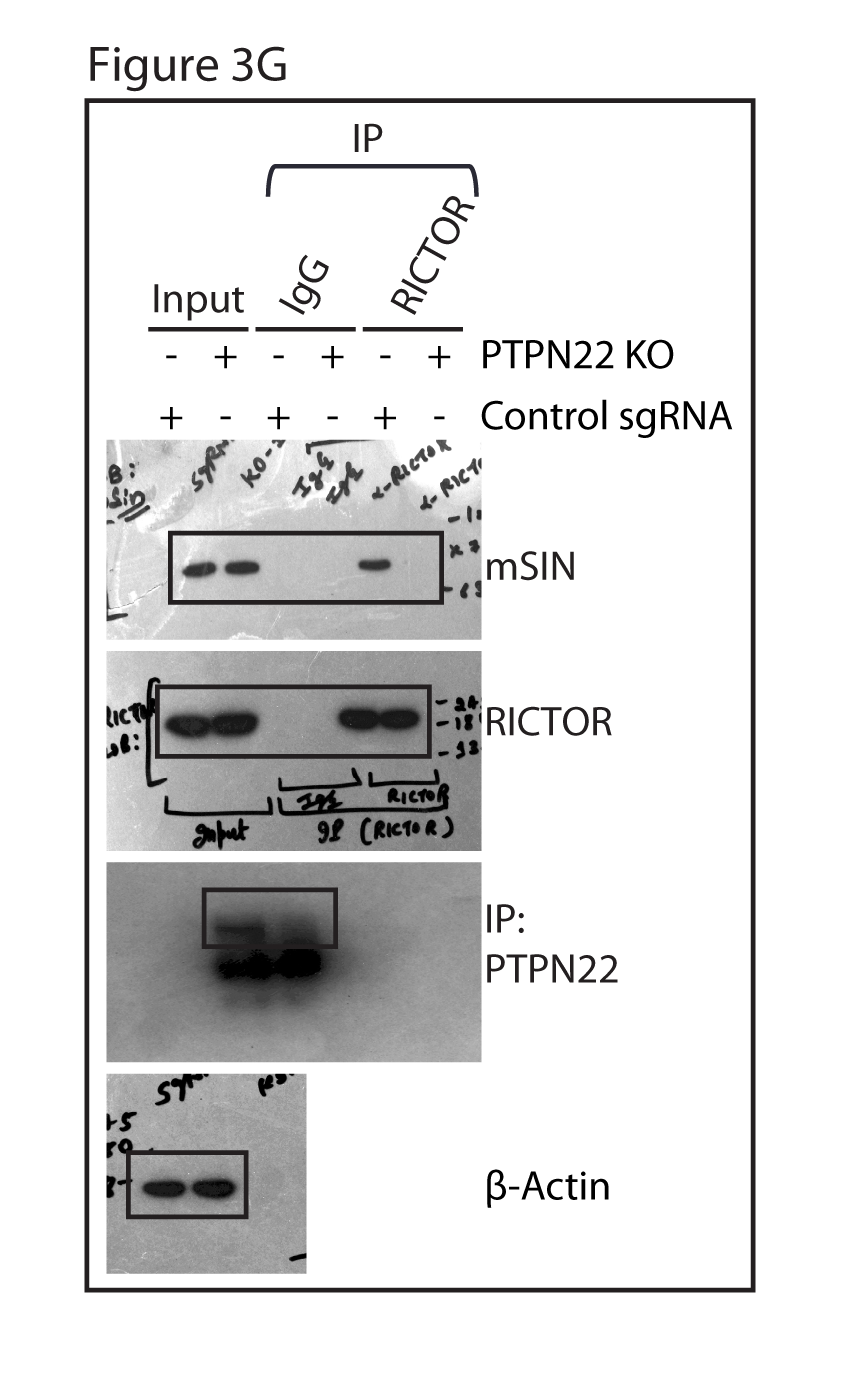

Supplement: Supplementary file 4 — Source data Fig. 3 [file 44319_2025_576_MOESM4_ESM.zip › Figure 3/3G/3G.tif]

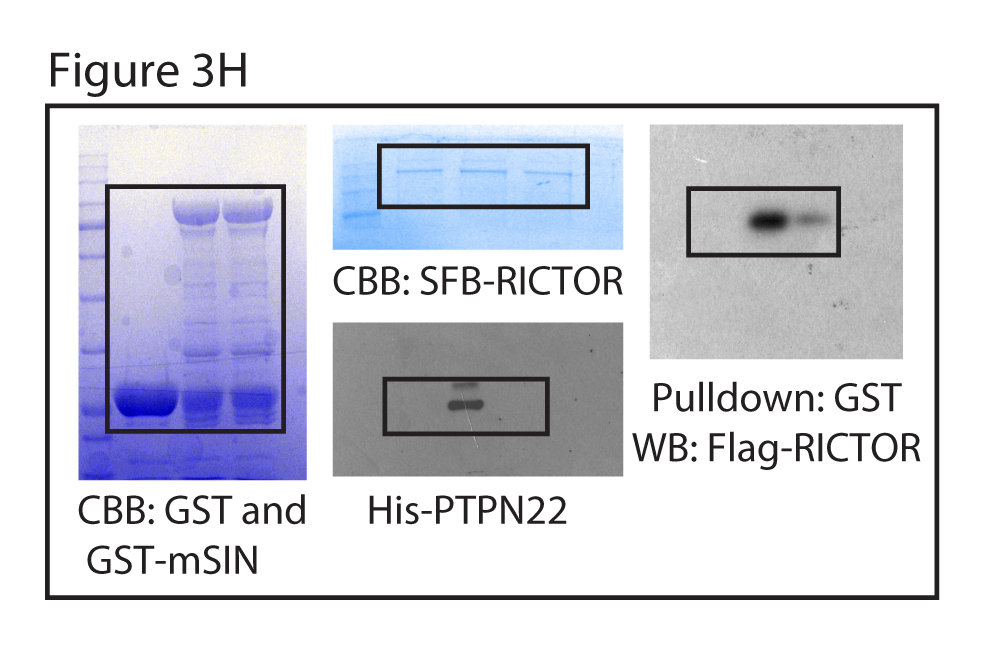

Supplement: Supplementary file 4 — Source data Fig. 3 [file 44319_2025_576_MOESM4_ESM.zip › Figure 3/3H/3H.tif]

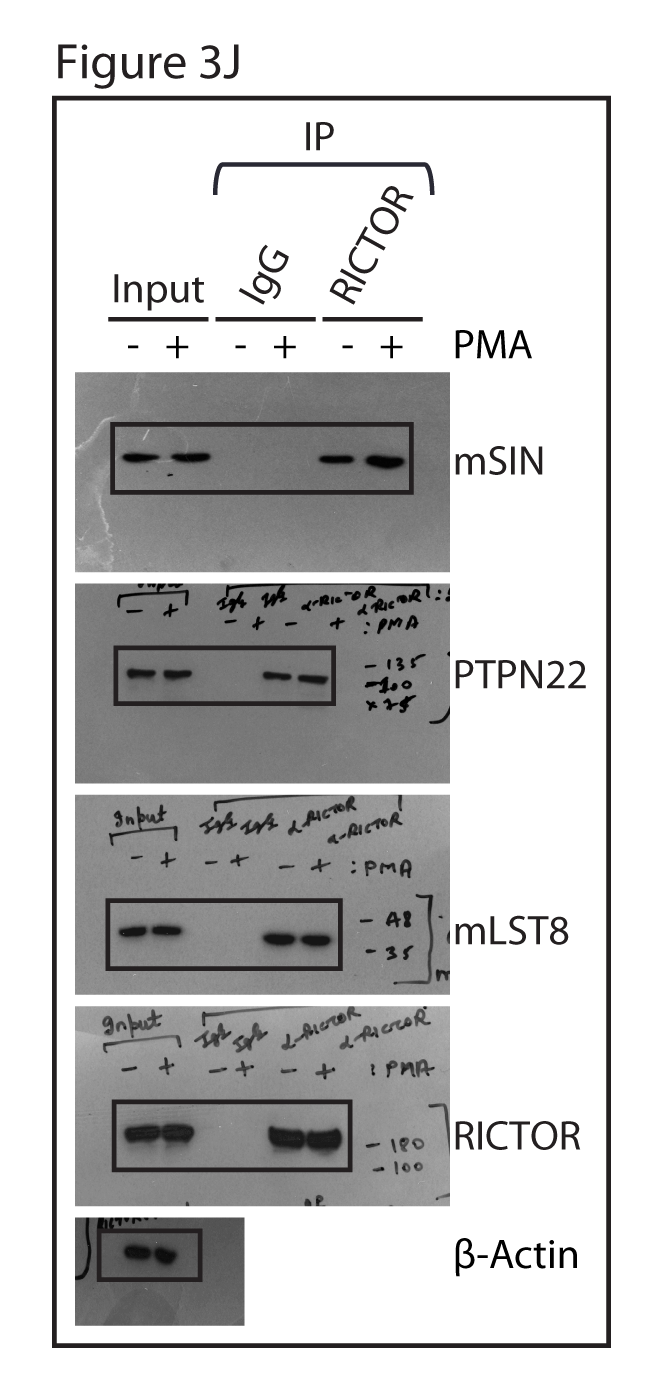

Supplement: Supplementary file 4 — Source data Fig. 3 [file 44319_2025_576_MOESM4_ESM.zip › Figure 3/3J/3J.tif]

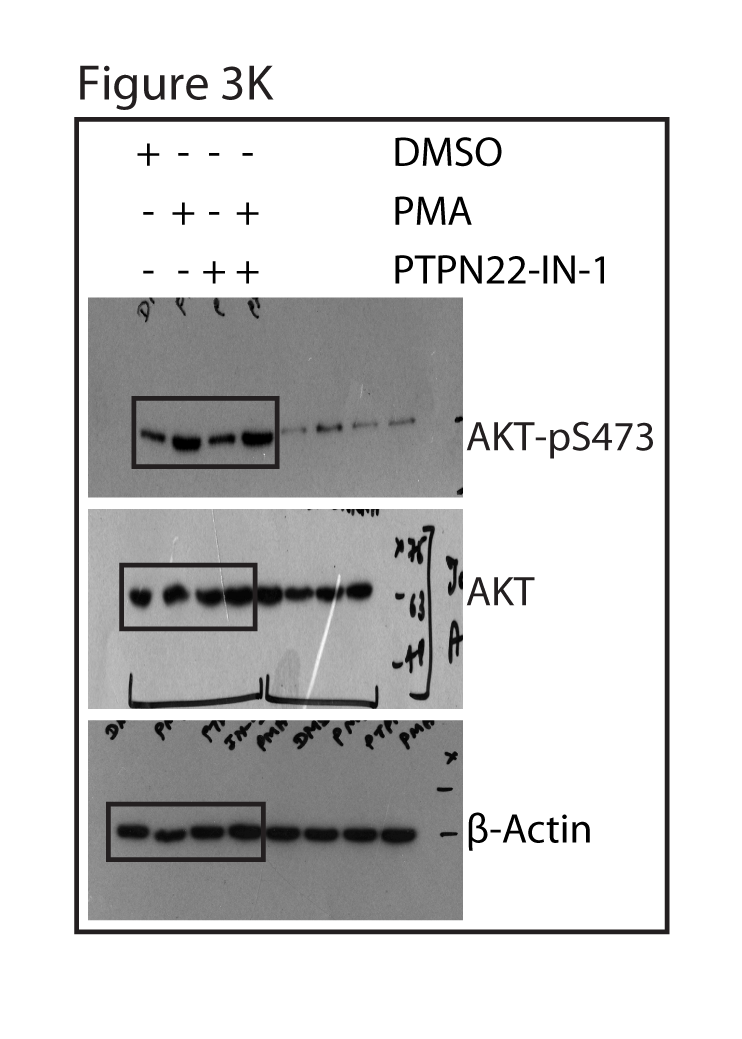

Supplement: Supplementary file 4 — Source data Fig. 3 [file 44319_2025_576_MOESM4_ESM.zip › Figure 3/3K/3K.tif]

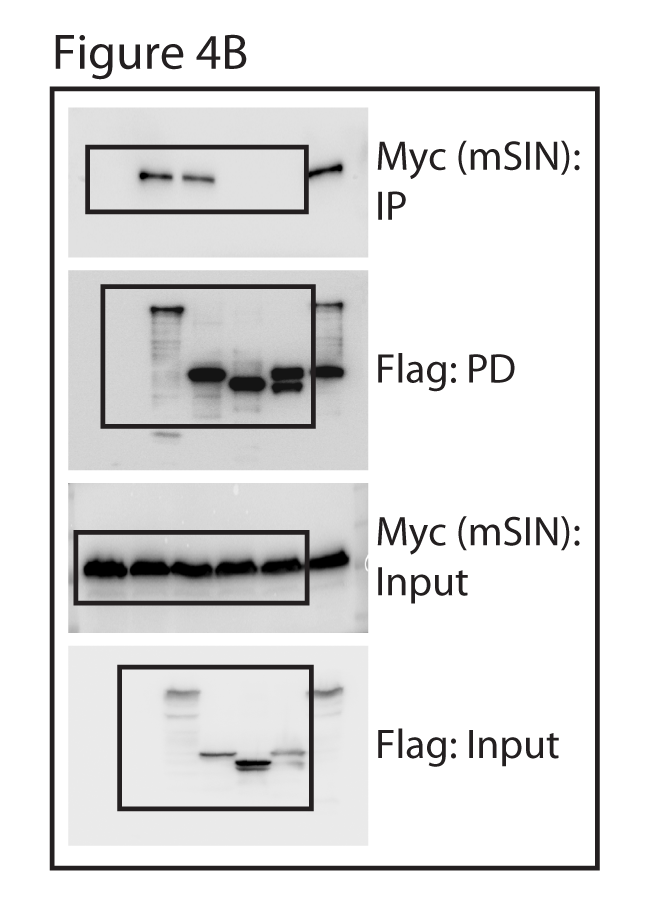

Supplement: Supplementary file 5 — Source data Fig. 4 [file 44319_2025_576_MOESM5_ESM.zip › Figure 4/4B/4B.tif]

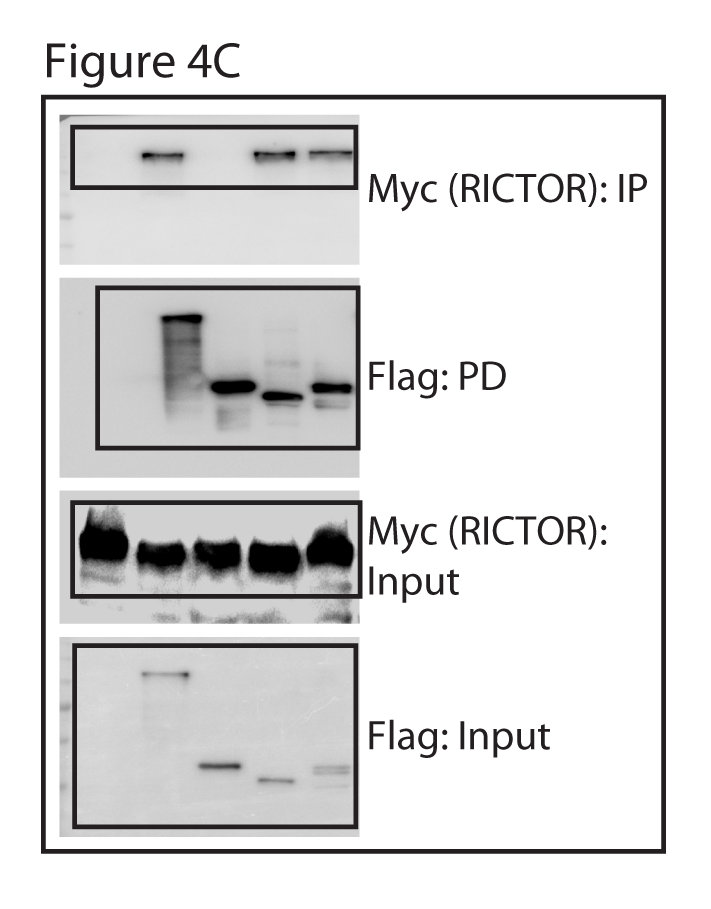

Supplement: Supplementary file 5 — Source data Fig. 4 [file 44319_2025_576_MOESM5_ESM.zip › Figure 4/4C/4C.tif]

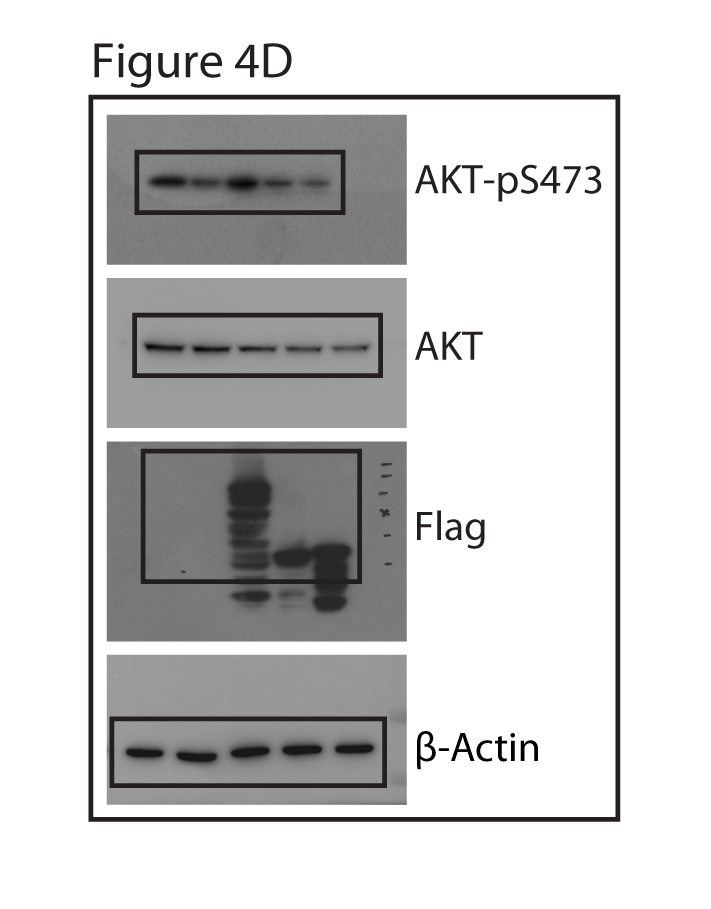

Supplement: Supplementary file 5 — Source data Fig. 4 [file 44319_2025_576_MOESM5_ESM.zip › Figure 4/4D/4D.tif]

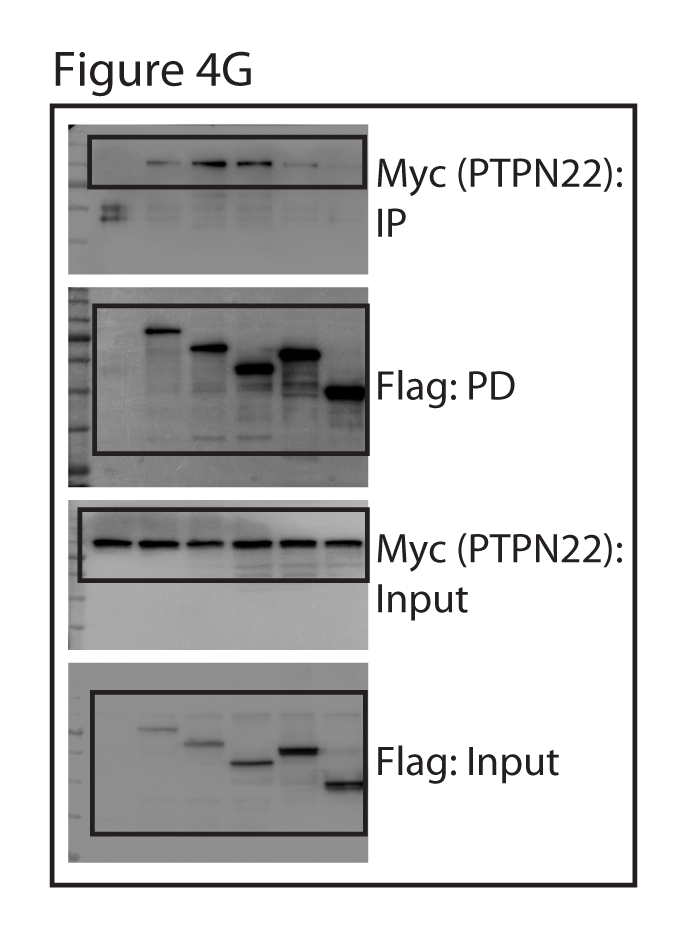

Supplement: Supplementary file 5 — Source data Fig. 4 [file 44319_2025_576_MOESM5_ESM.zip › Figure 4/4G/4G.tif]

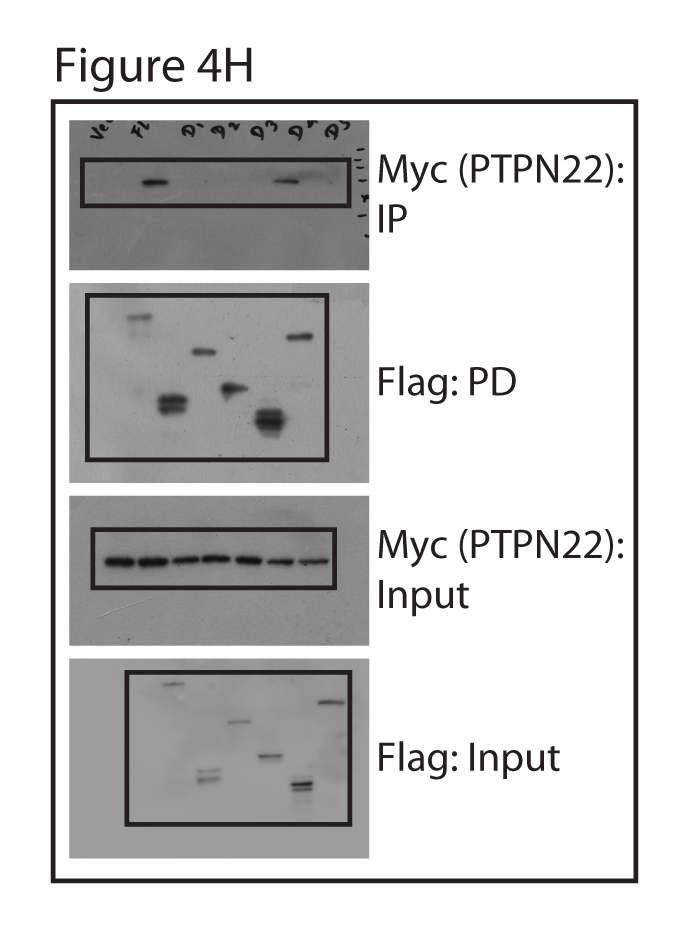

Supplement: Supplementary file 5 — Source data Fig. 4 [file 44319_2025_576_MOESM5_ESM.zip › Figure 4/4H/4H.tif]

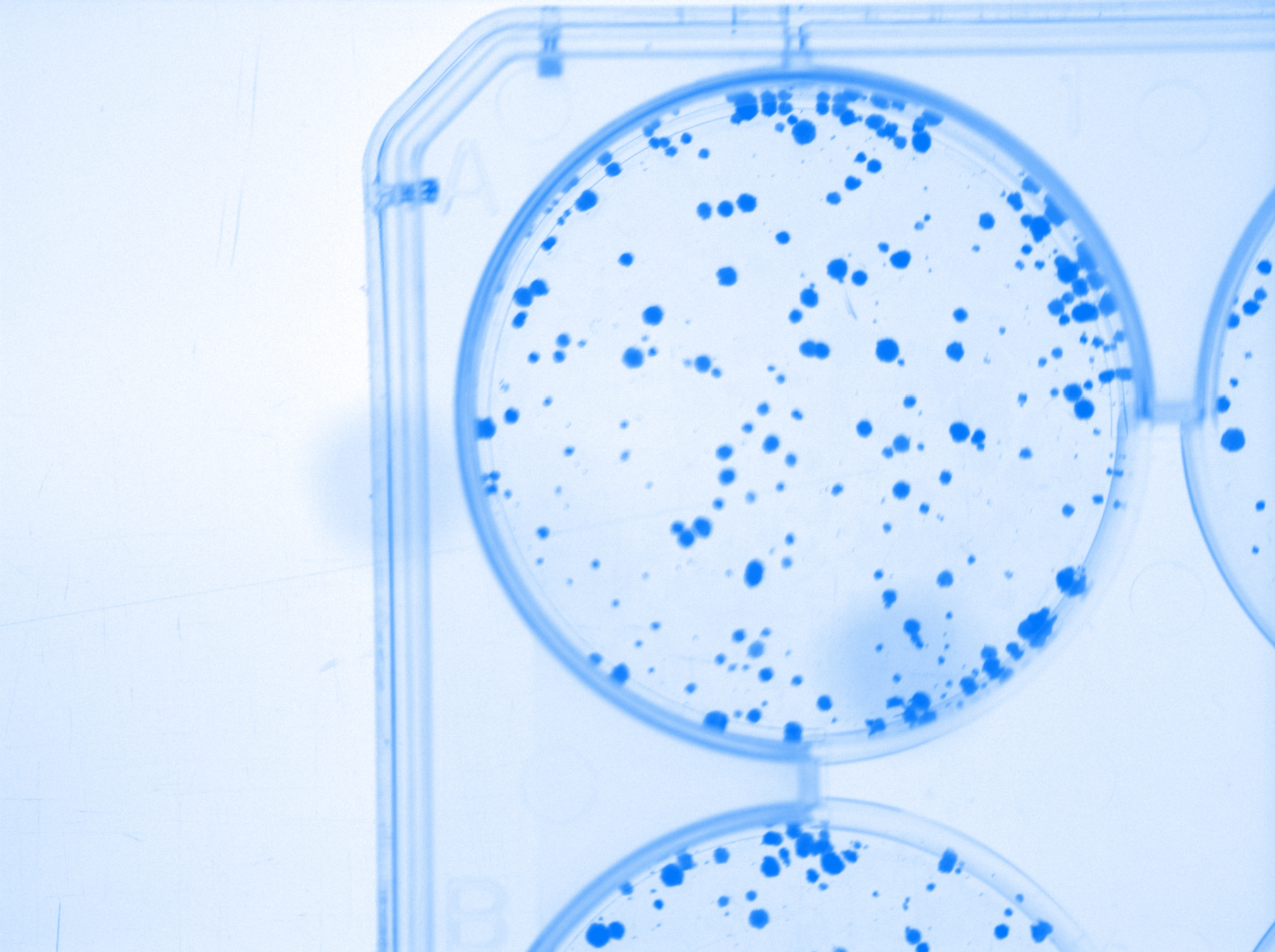

Supplement: Supplementary file 6 — Source data Fig. 5 [file 44319_2025_576_MOESM6_ESM.zip › Figure 5/5B/Control shRNA + Empty Vector.tif]

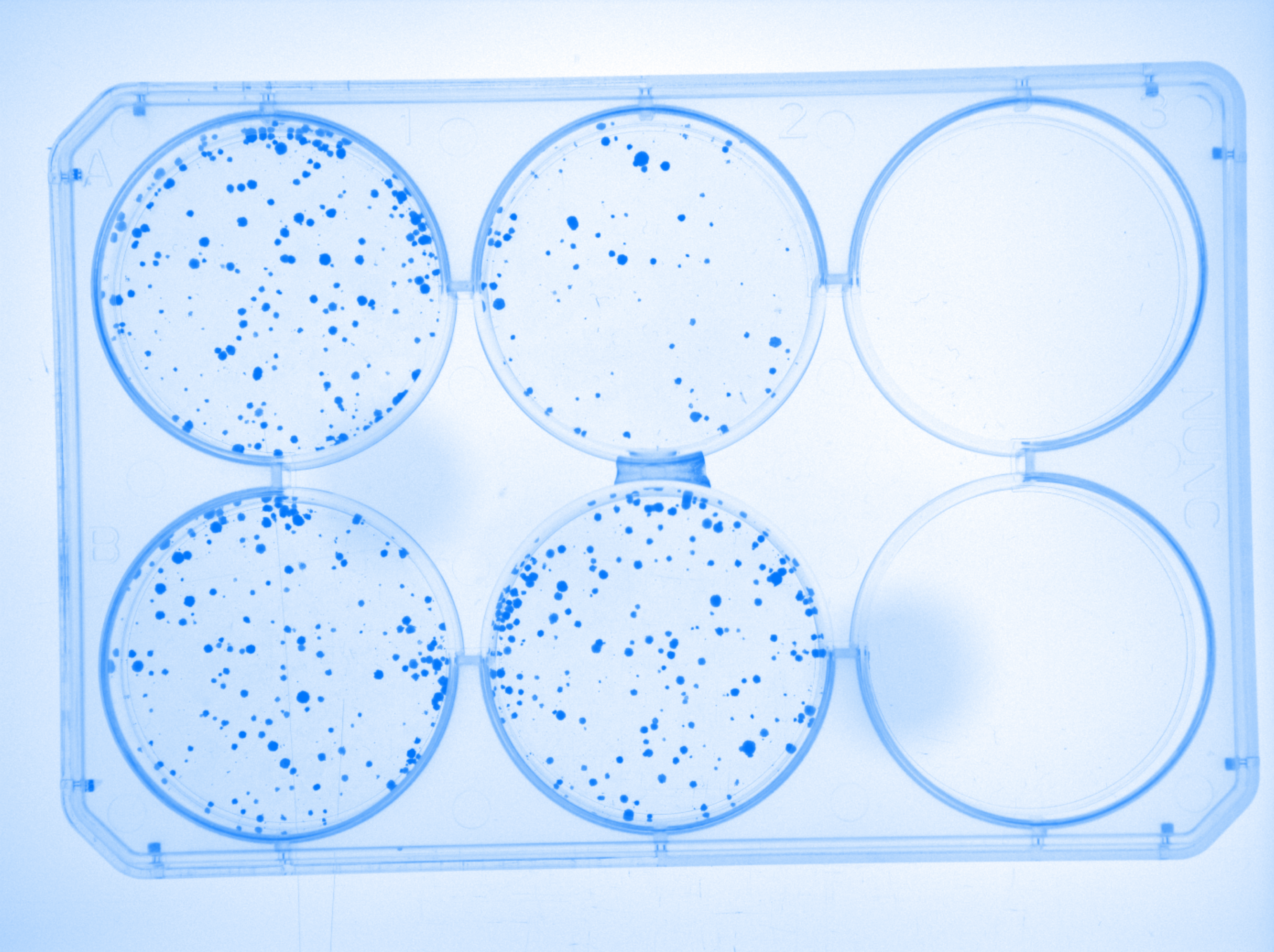

Supplement: Supplementary file 6 — Source data Fig. 5 [file 44319_2025_576_MOESM6_ESM.zip › Figure 5/5B/Full Plate_All Samples Together.tif]

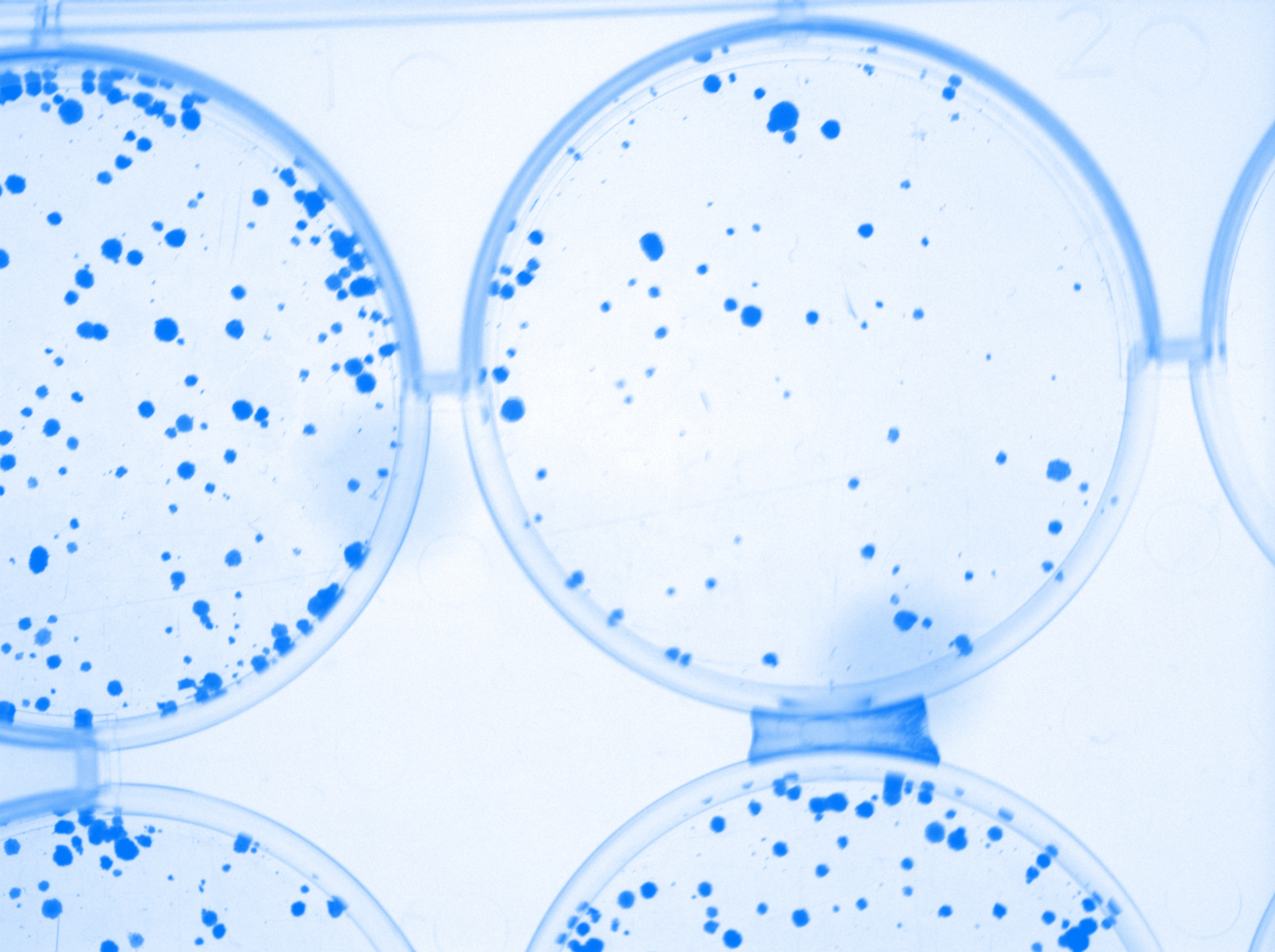

Supplement: Supplementary file 6 — Source data Fig. 5 [file 44319_2025_576_MOESM6_ESM.zip › Figure 5/5B/PTPN22 shRNA-2 + Empty Vector.tif]

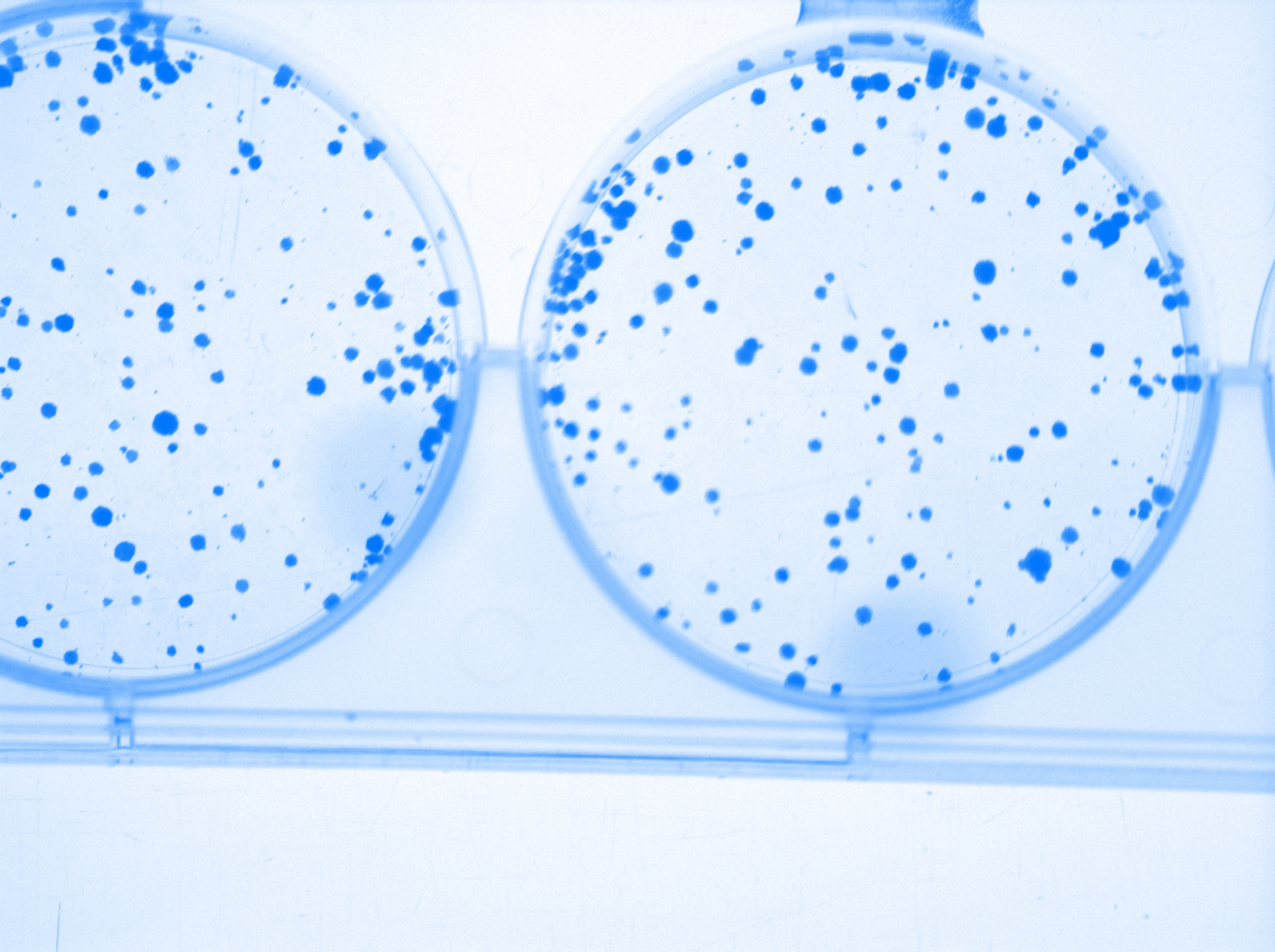

Supplement: Supplementary file 6 — Source data Fig. 5 [file 44319_2025_576_MOESM6_ESM.zip › Figure 5/5B/PTPN22 shRNA-2 + PTPN22 (D_A-C_S) shRNA Res..tif]

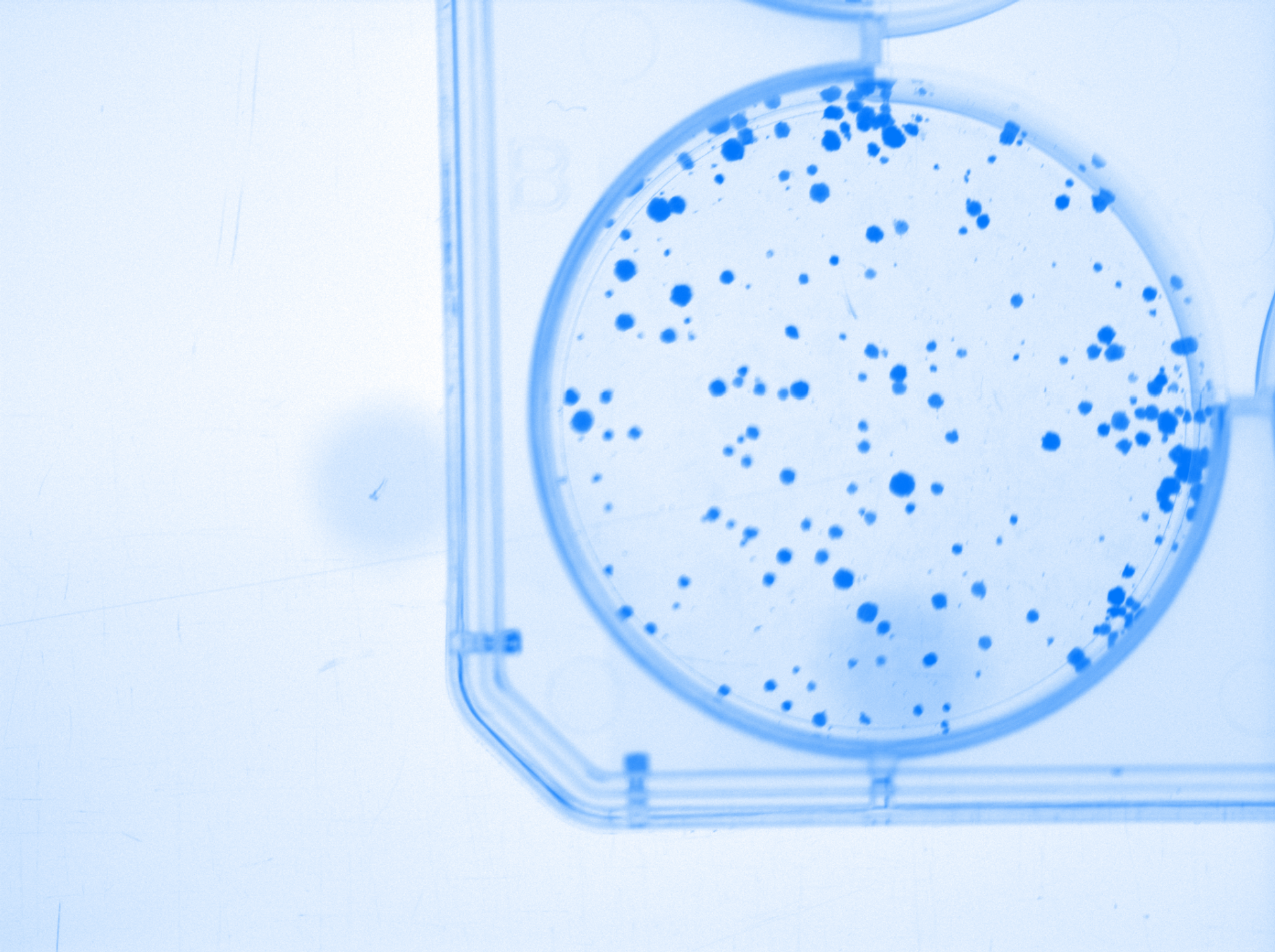

Supplement: Supplementary file 6 — Source data Fig. 5 [file 44319_2025_576_MOESM6_ESM.zip › Figure 5/5B/PTPN22 shRNA-2 + PTPN22 (WT) shRNA Res..tif]

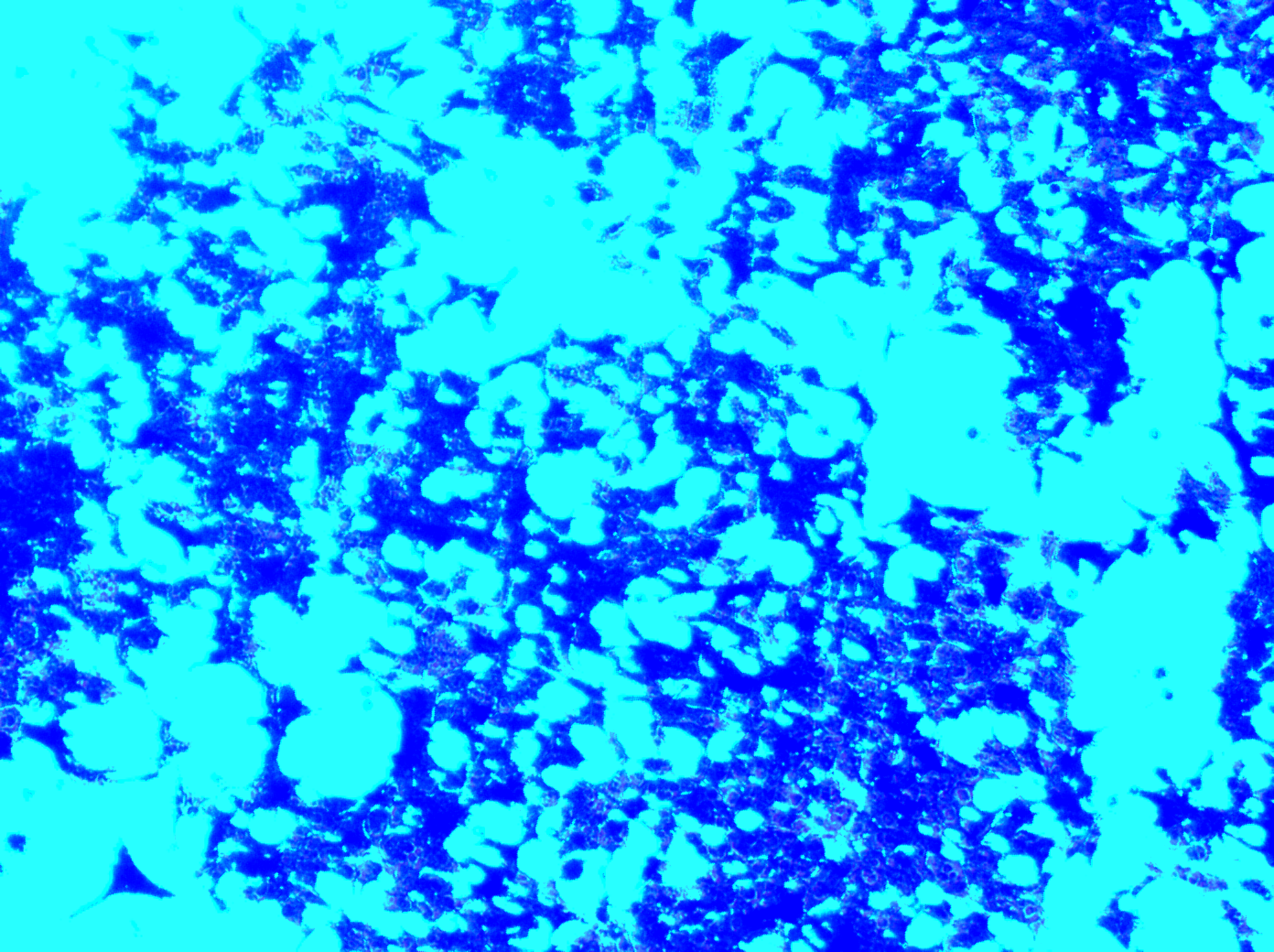

Supplement: Supplementary file 6 — Source data Fig. 5 [file 44319_2025_576_MOESM6_ESM.zip › Figure 5/5D/Control shRNA.tif]

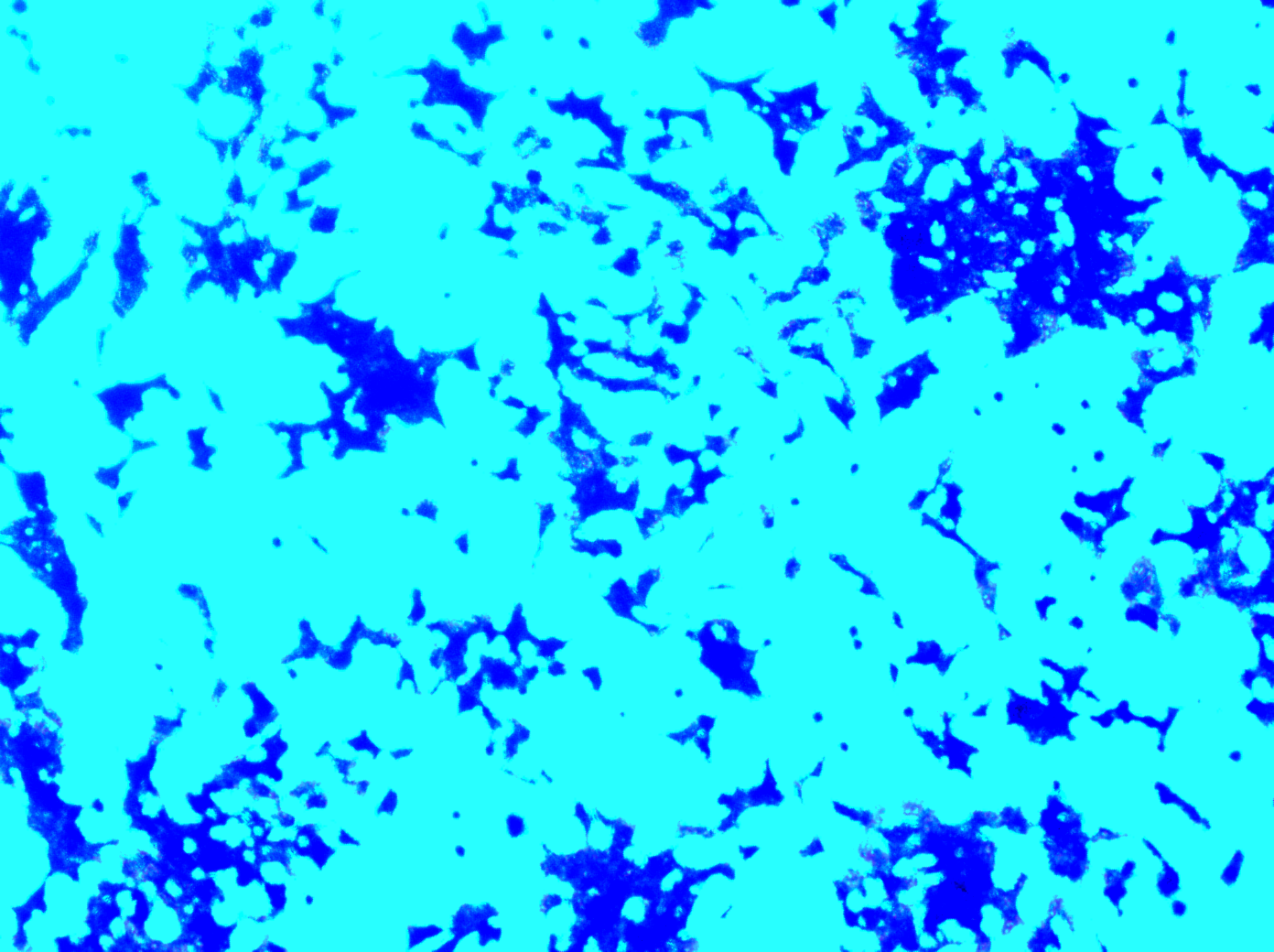

Supplement: Supplementary file 6 — Source data Fig. 5 [file 44319_2025_576_MOESM6_ESM.zip › Figure 5/5D/PTPN22 shRNA-1.tif]

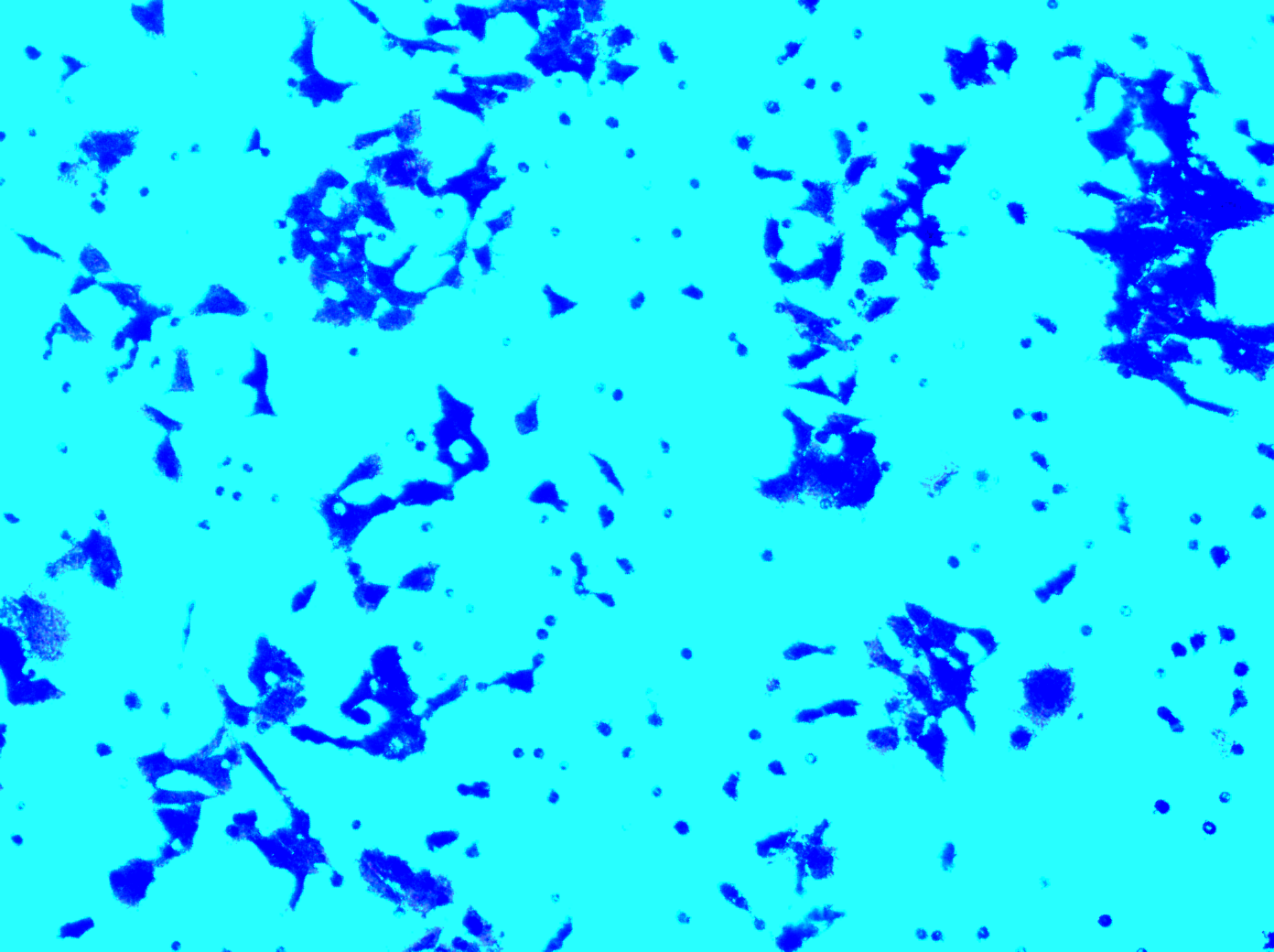

Supplement: Supplementary file 6 — Source data Fig. 5 [file 44319_2025_576_MOESM6_ESM.zip › Figure 5/5D/PTPN22 shRNA-2 + Empty Vector.tif]

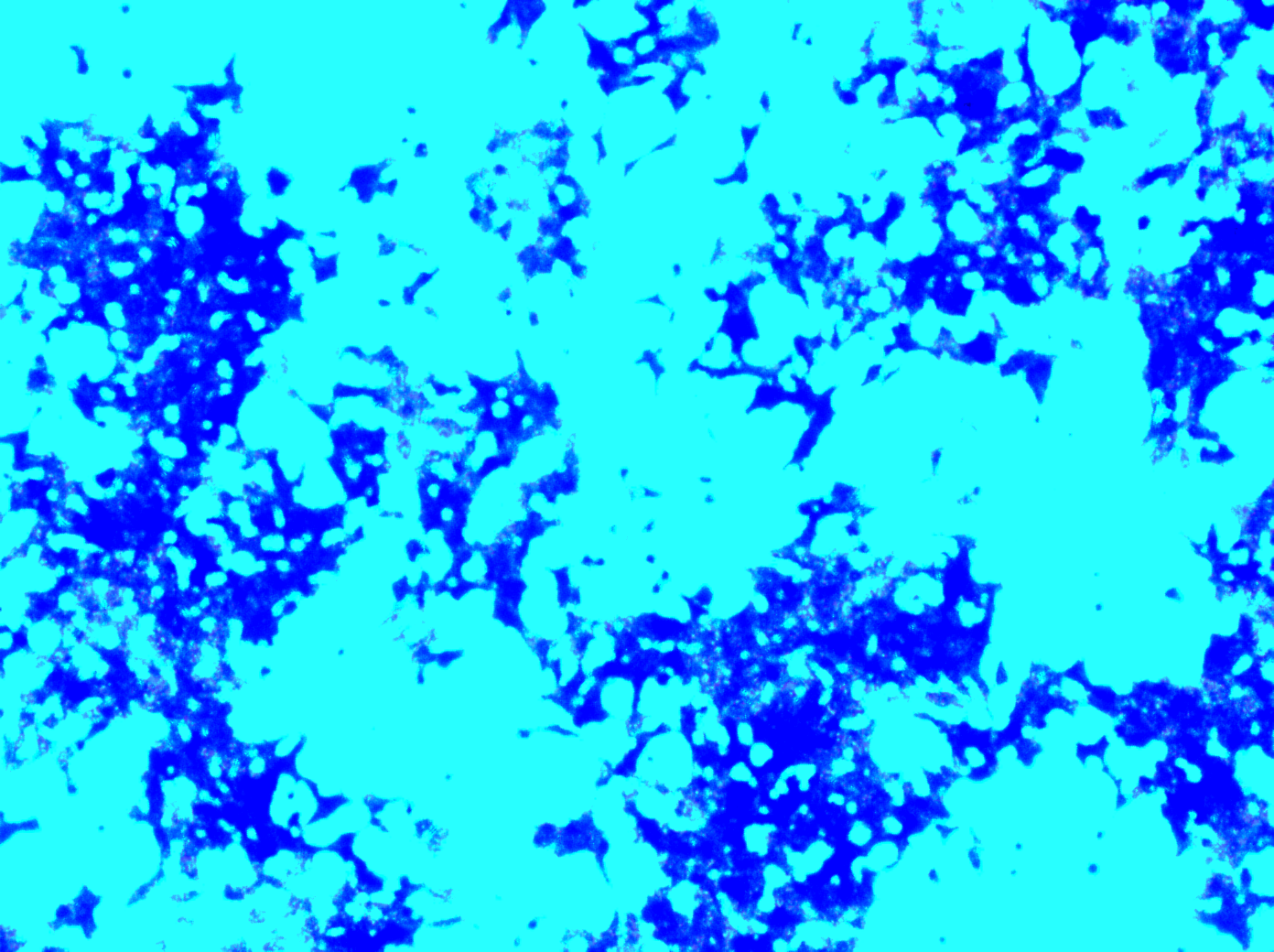

Supplement: Supplementary file 6 — Source data Fig. 5 [file 44319_2025_576_MOESM6_ESM.zip › Figure 5/5D/PTPN22 shRNA-2 + PTPN22 (D_A-C_S) shRNA Res..tif]

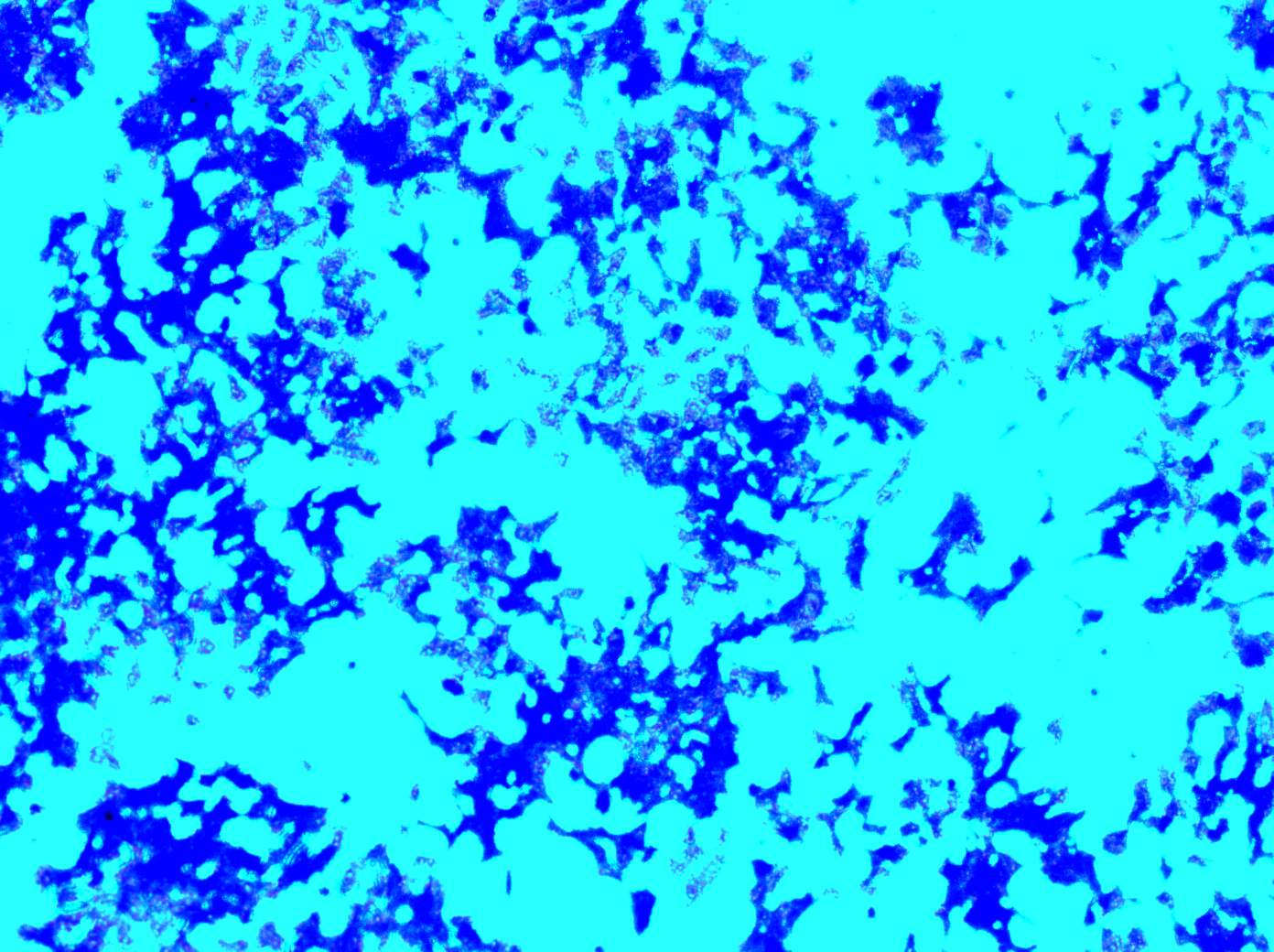

Supplement: Supplementary file 6 — Source data Fig. 5 [file 44319_2025_576_MOESM6_ESM.zip › Figure 5/5D/PTPN22 shRNA-2 + PTPN22 (WT) shRNA Res..tif]

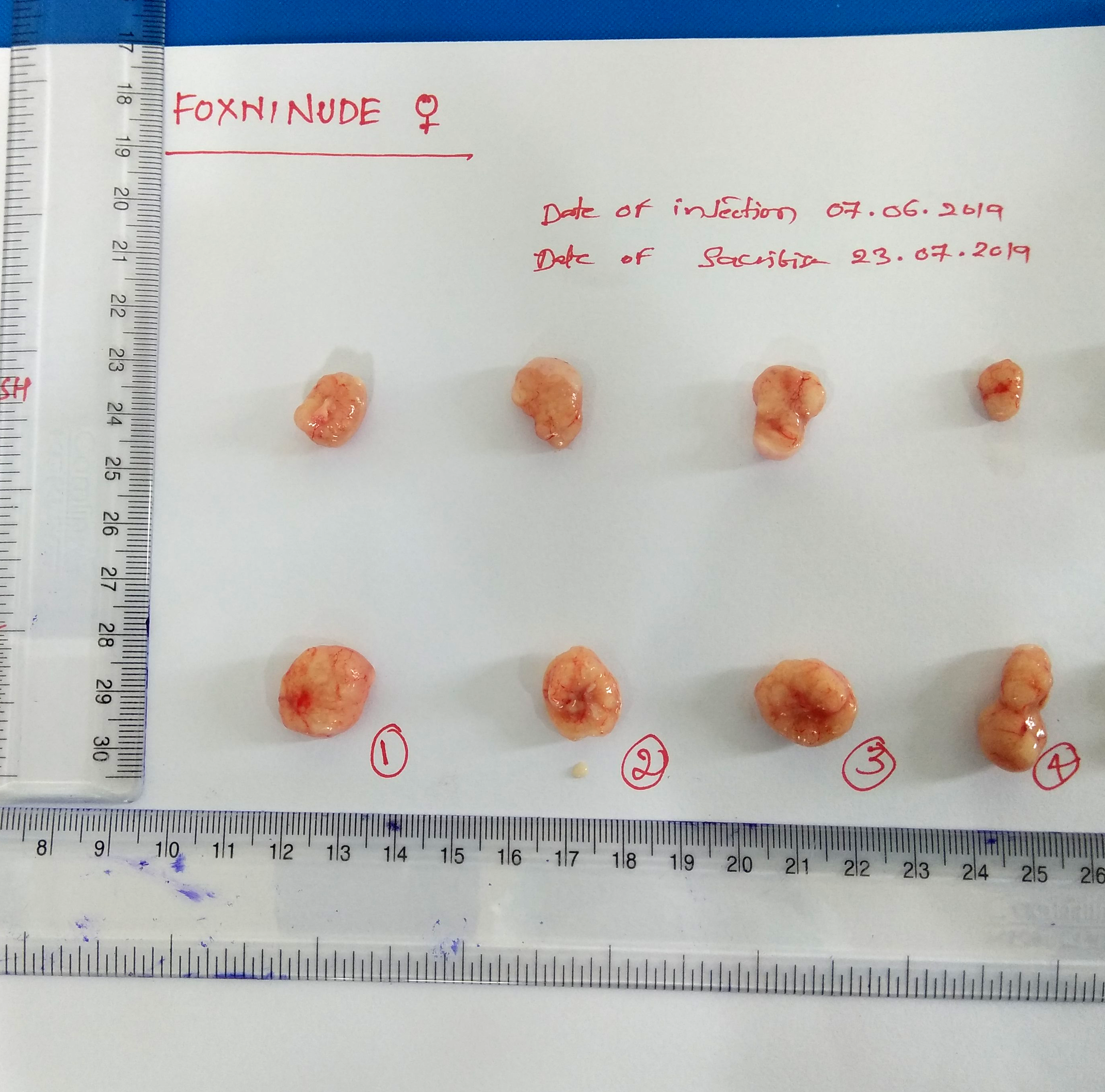

Supplement: Supplementary file 6 — Source data Fig. 5 [file 44319_2025_576_MOESM6_ESM.zip › Figure 5/5F/Tumor Image.tif]

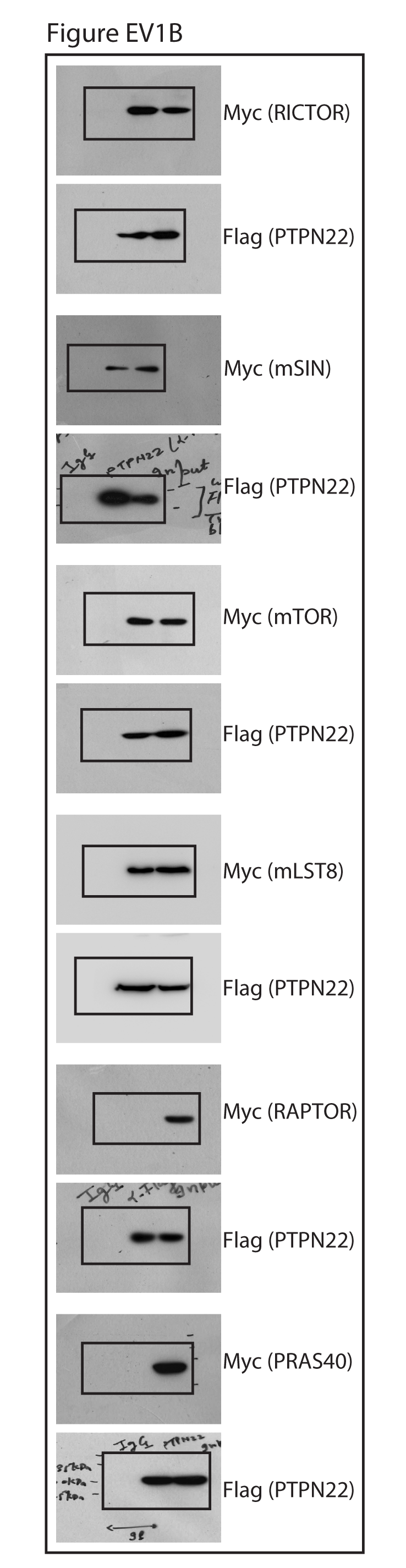

Supplement: Supplementary file 7 — Figure EV1 Source Data [file 44319_2025_576_MOESM7_ESM.zip › Figure EV1/EV1B/EV1B.tif]

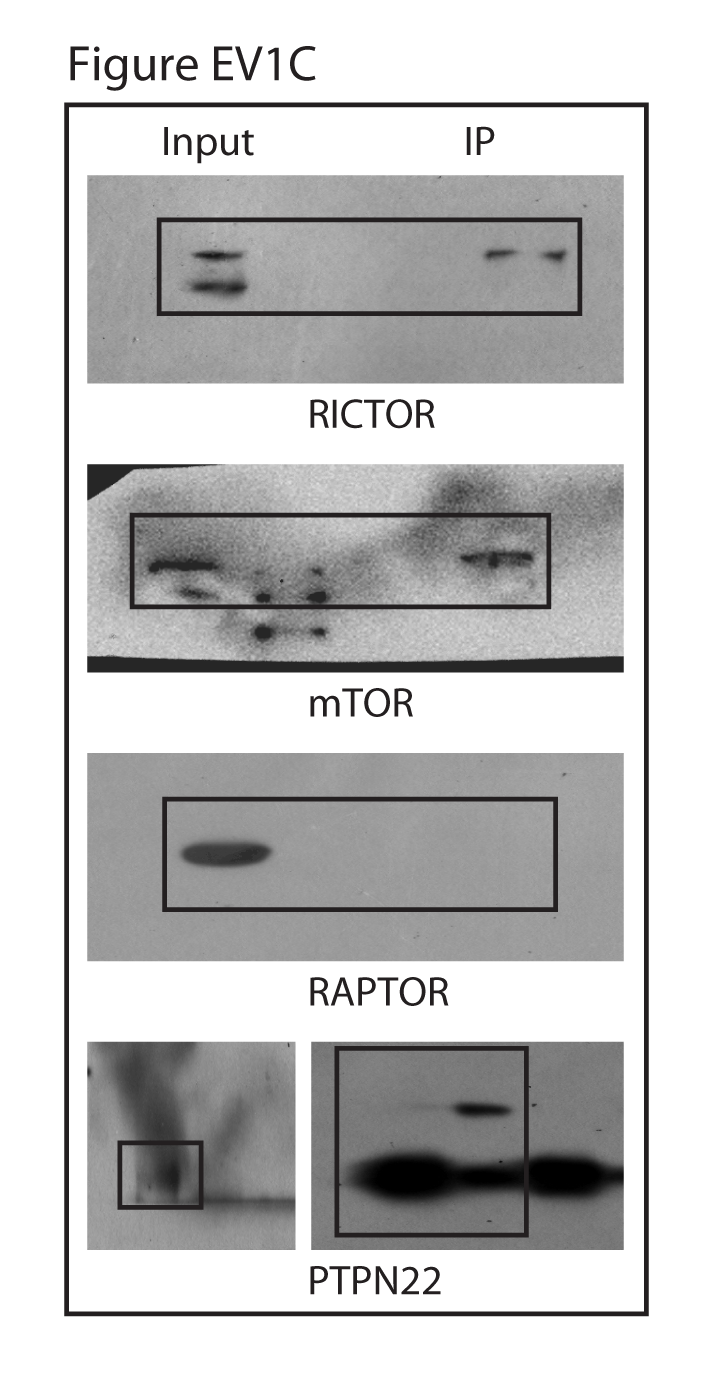

Supplement: Supplementary file 7 — Figure EV1 Source Data [file 44319_2025_576_MOESM7_ESM.zip › Figure EV1/EV1C/EV1C.tif]

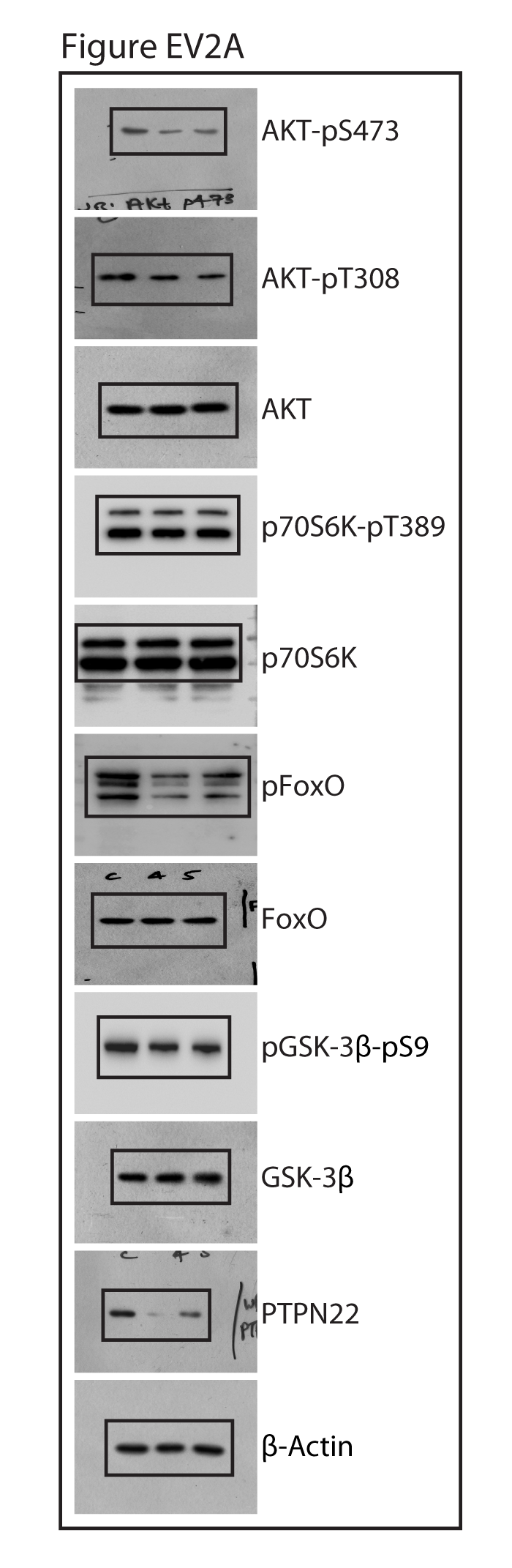

Supplement: Supplementary file 8 — Figure EV2 Source Data [file 44319_2025_576_MOESM8_ESM.zip › Figure EV2/EV2A/EV2A.tif]

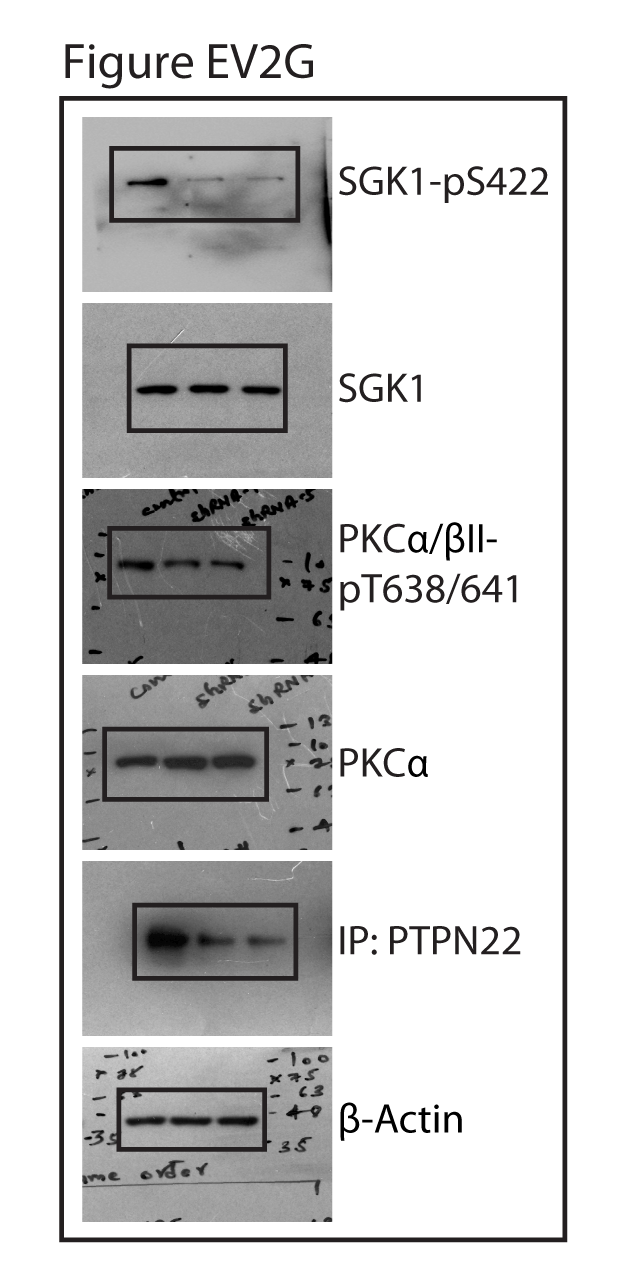

Supplement: Supplementary file 8 — Figure EV2 Source Data [file 44319_2025_576_MOESM8_ESM.zip › Figure EV2/EV2G/EV2G.tif]

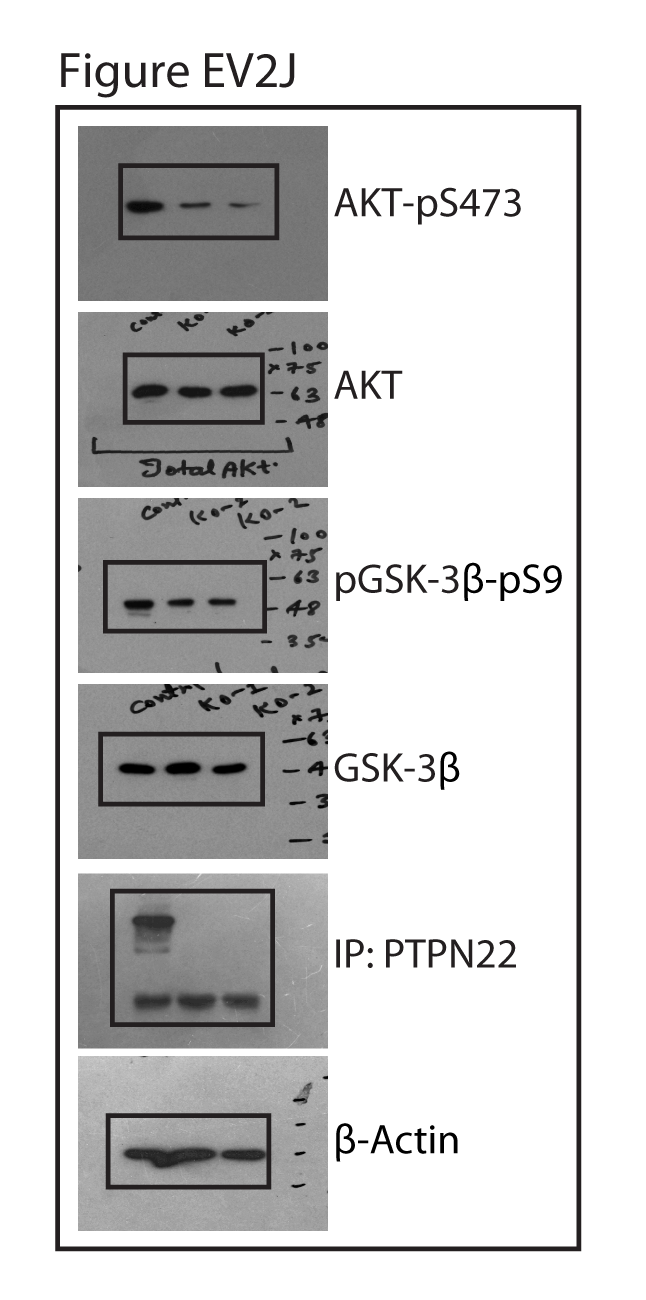

Supplement: Supplementary file 8 — Figure EV2 Source Data [file 44319_2025_576_MOESM8_ESM.zip › Figure EV2/EV2J/EV2J.tif]

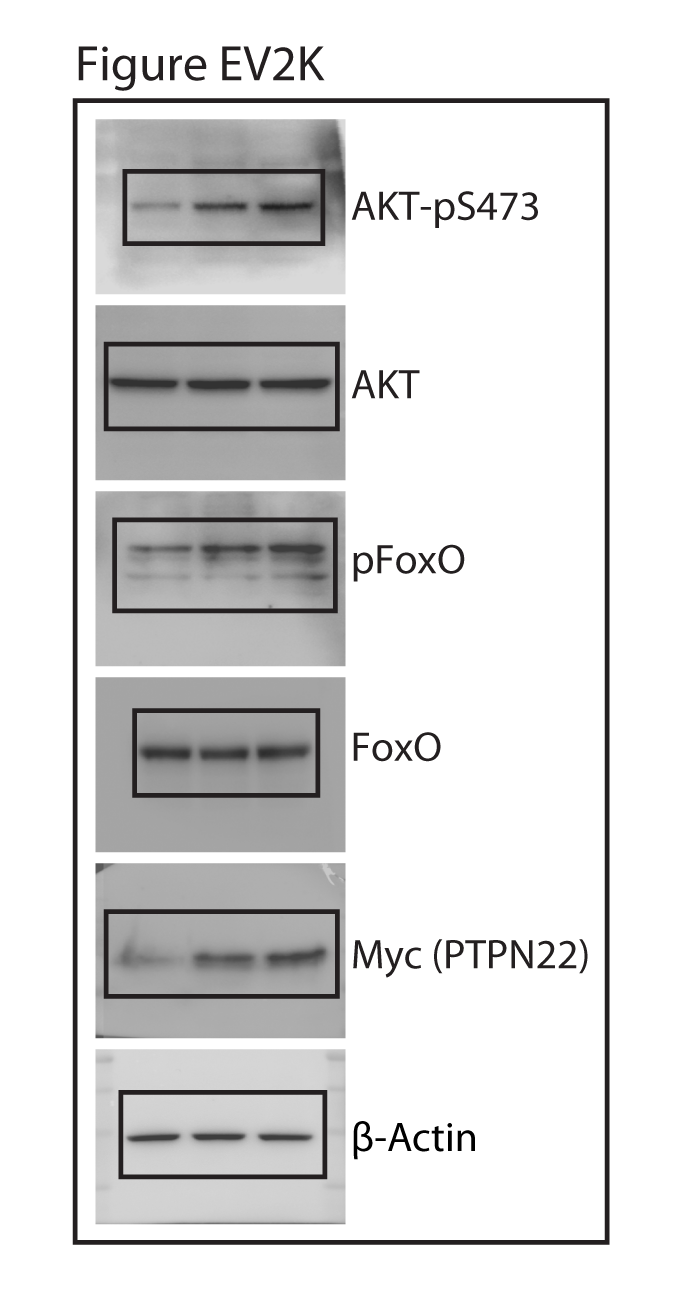

Supplement: Supplementary file 8 — Figure EV2 Source Data [file 44319_2025_576_MOESM8_ESM.zip › Figure EV2/EV2K/EV2K.tif]

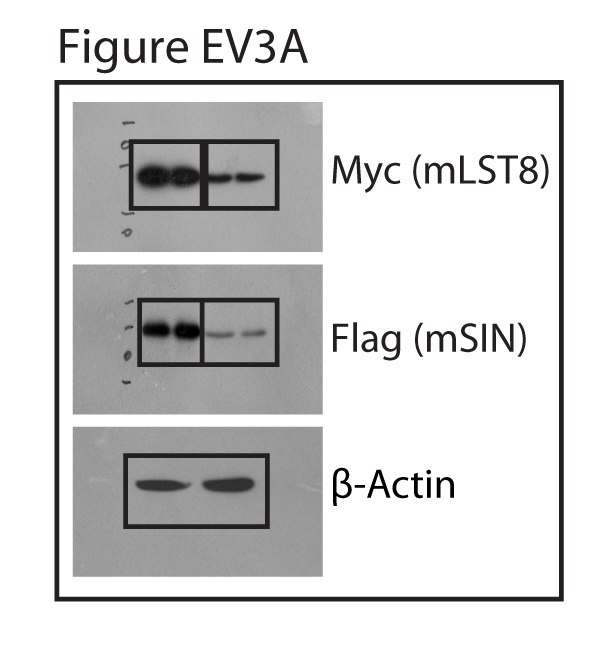

Supplement: Supplementary file 9 — Figure EV3 Source Data [file 44319_2025_576_MOESM9_ESM.zip › Figure EV3/EV3A/EV3A.tif]

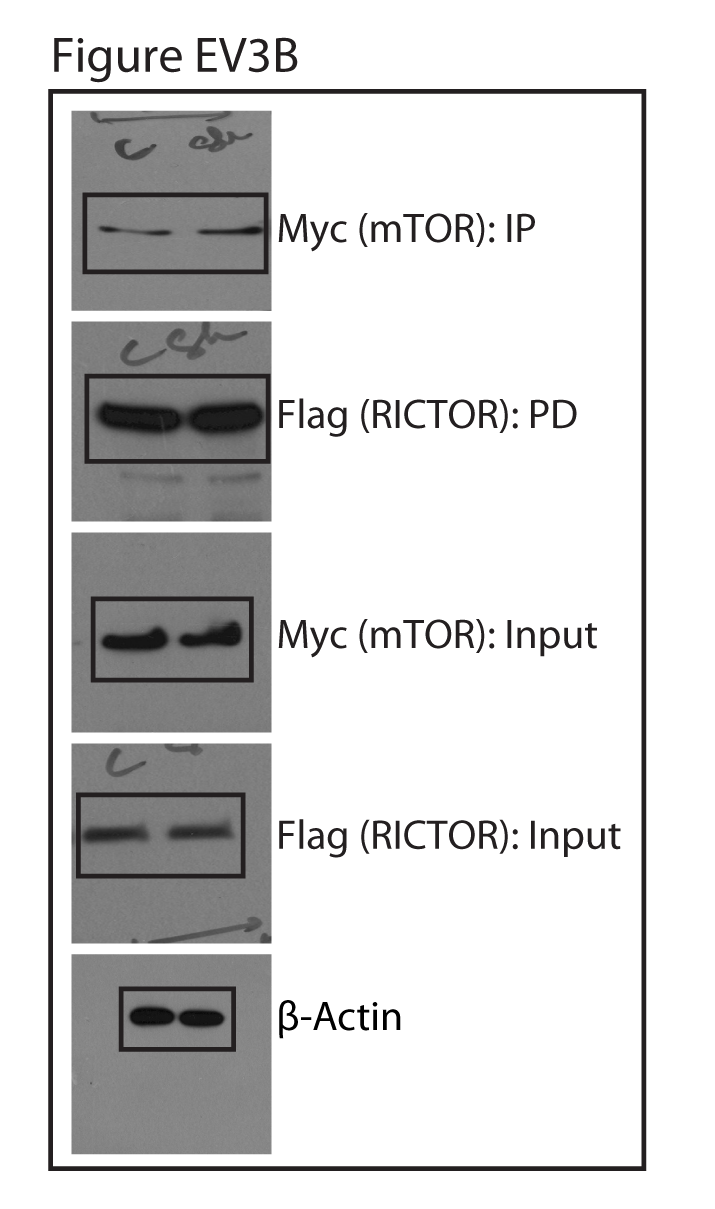

Supplement: Supplementary file 9 — Figure EV3 Source Data [file 44319_2025_576_MOESM9_ESM.zip › Figure EV3/EV3B/EV3B.tif]

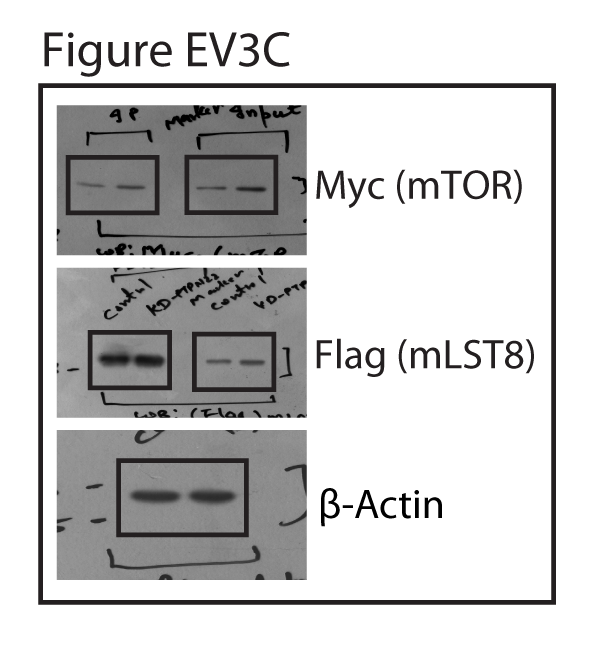

Supplement: Supplementary file 9 — Figure EV3 Source Data [file 44319_2025_576_MOESM9_ESM.zip › Figure EV3/EV3C/EV3C.tif]

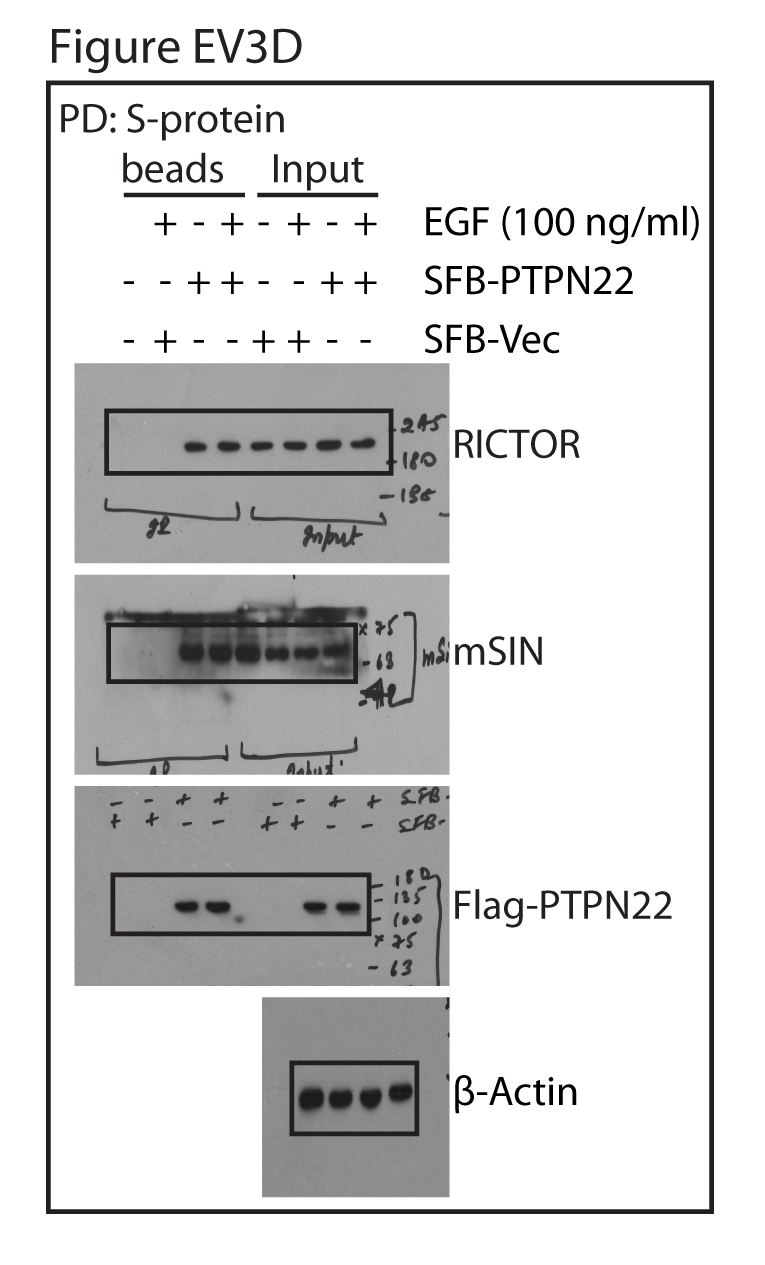

Supplement: Supplementary file 9 — Figure EV3 Source Data [file 44319_2025_576_MOESM9_ESM.zip › Figure EV3/EV3D/EV3D.tif]

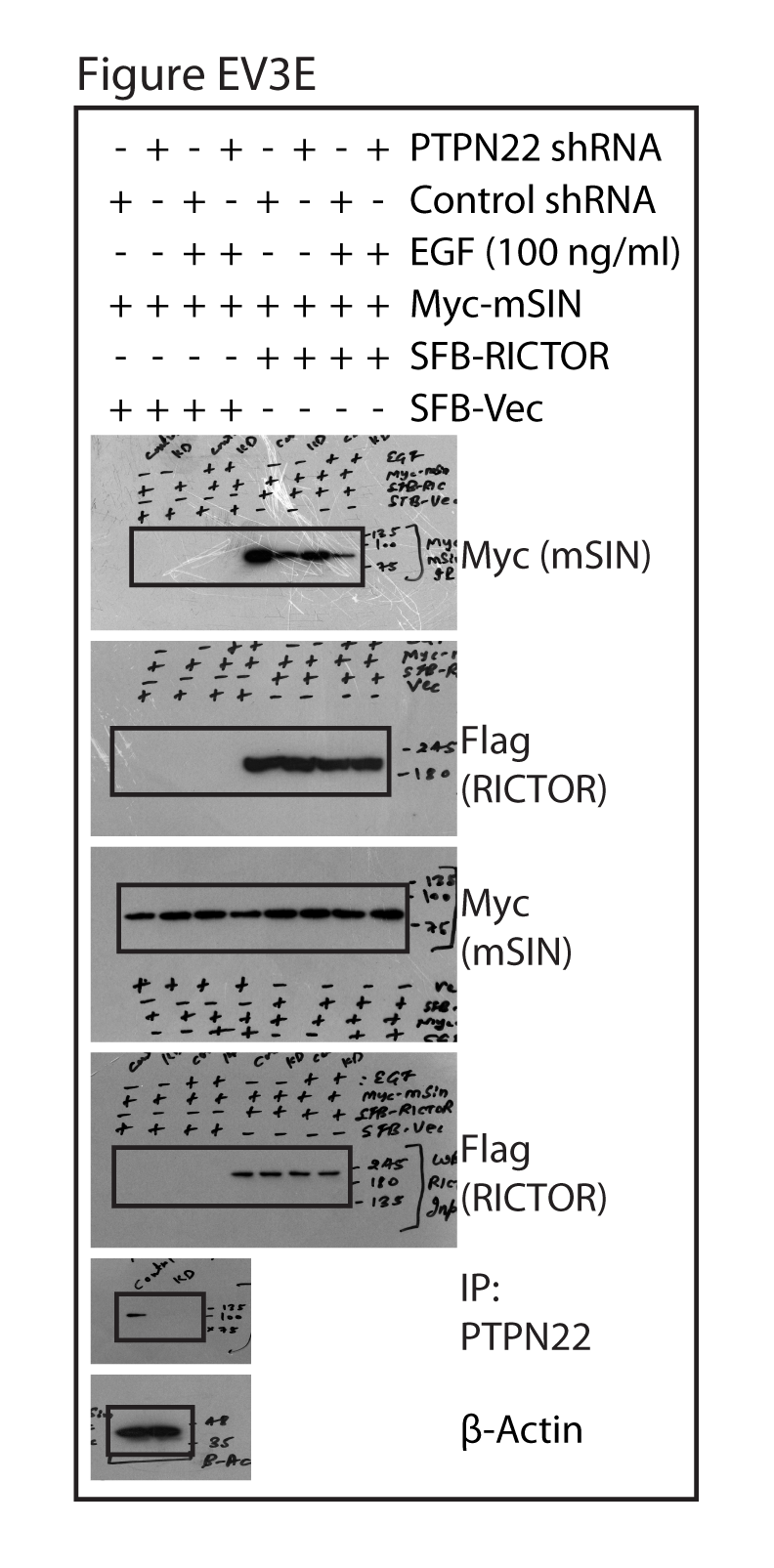

Supplement: Supplementary file 9 — Figure EV3 Source Data [file 44319_2025_576_MOESM9_ESM.zip › Figure EV3/EV3E/EV3E.tif]

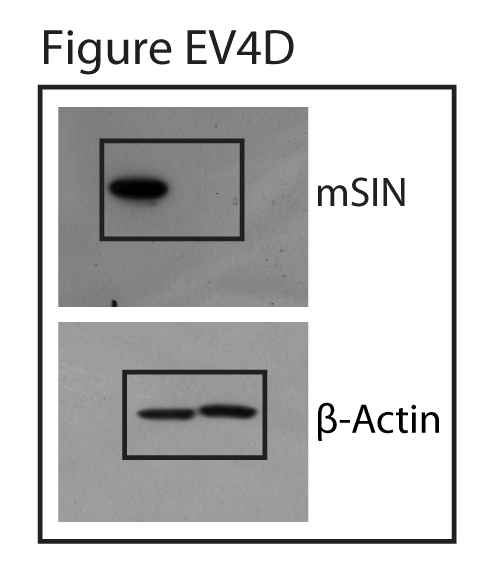

Supplement: Supplementary file 10 — Figure EV4 Source Data [file 44319_2025_576_MOESM10_ESM.zip › Figure EV4/EV4D/EV4D.tif]

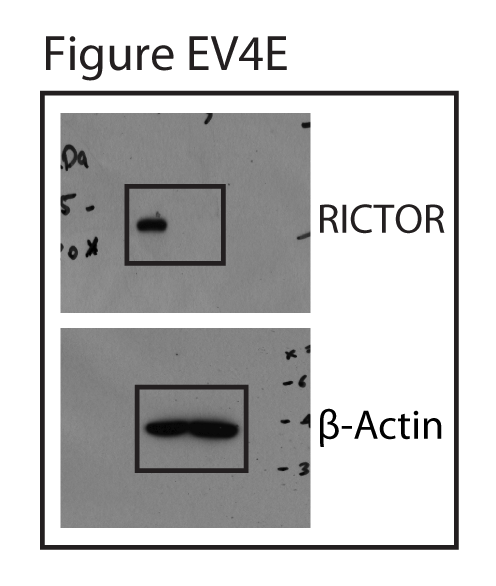

Supplement: Supplementary file 10 — Figure EV4 Source Data [file 44319_2025_576_MOESM10_ESM.zip › Figure EV4/EV4E/EV4E.tif]

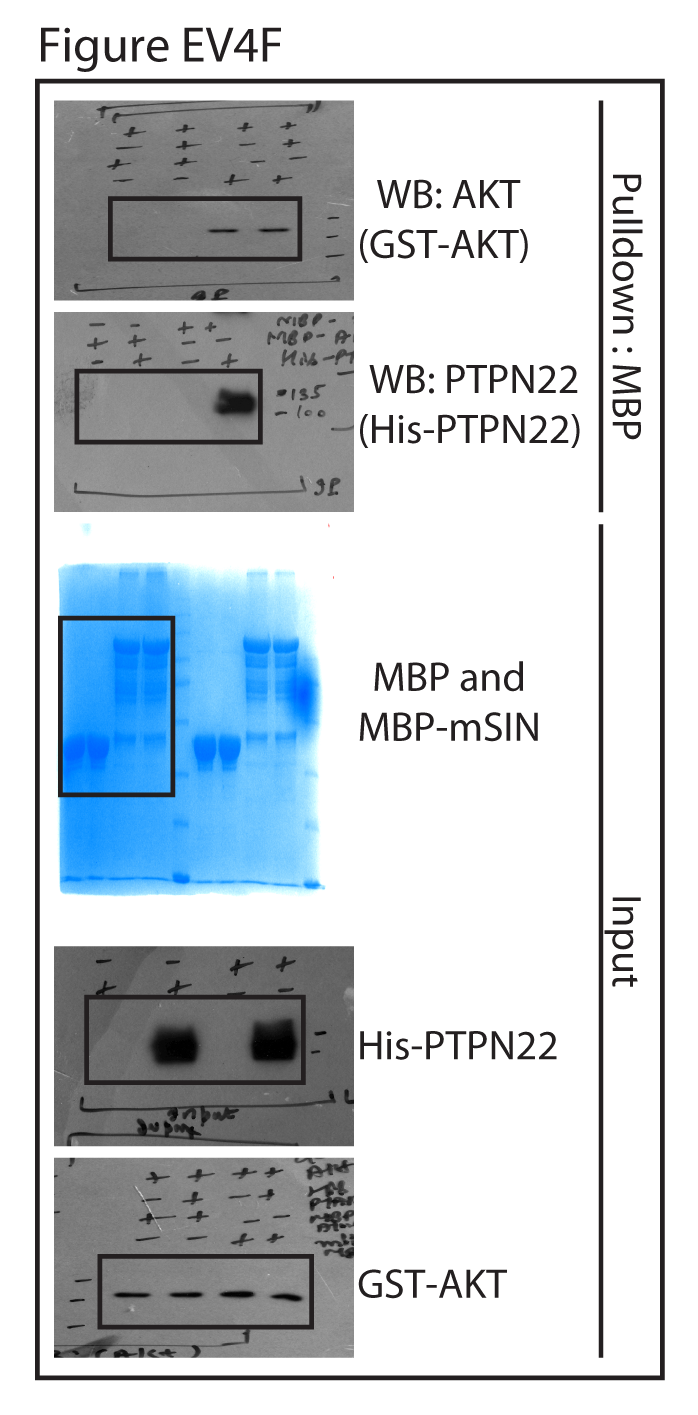

Supplement: Supplementary file 10 — Figure EV4 Source Data [file 44319_2025_576_MOESM10_ESM.zip › Figure EV4/EV4F/EV4F.tif]

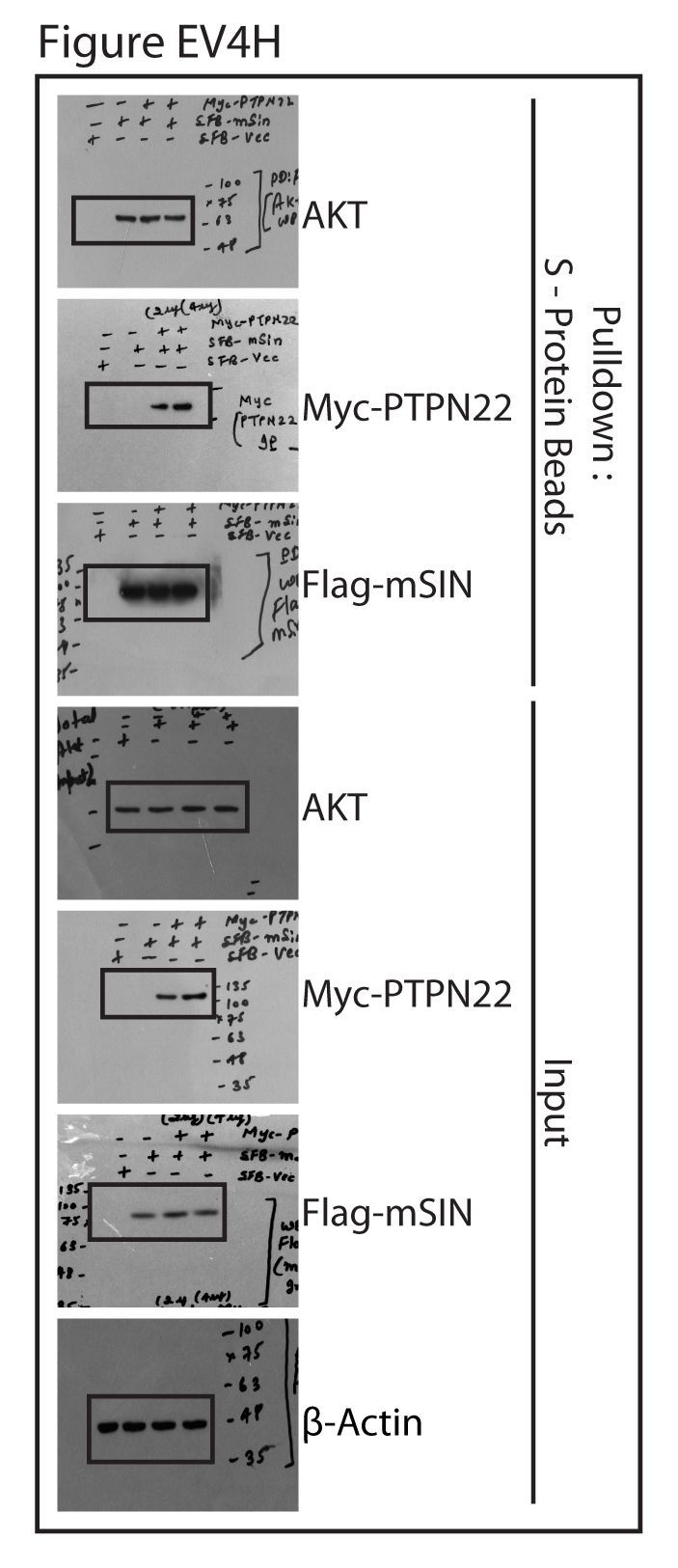

Supplement: Supplementary file 10 — Figure EV4 Source Data [file 44319_2025_576_MOESM10_ESM.zip › Figure EV4/EV4H/EV4H.tif]

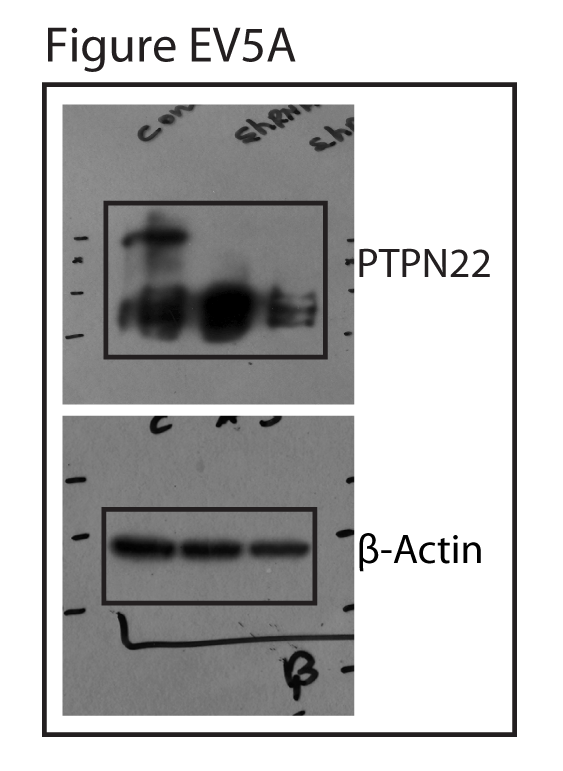

Supplement: Supplementary file 11 — Figure EV5 Source Data [file 44319_2025_576_MOESM11_ESM.zip › Figure EV5/EV5A/EV5A.tif]

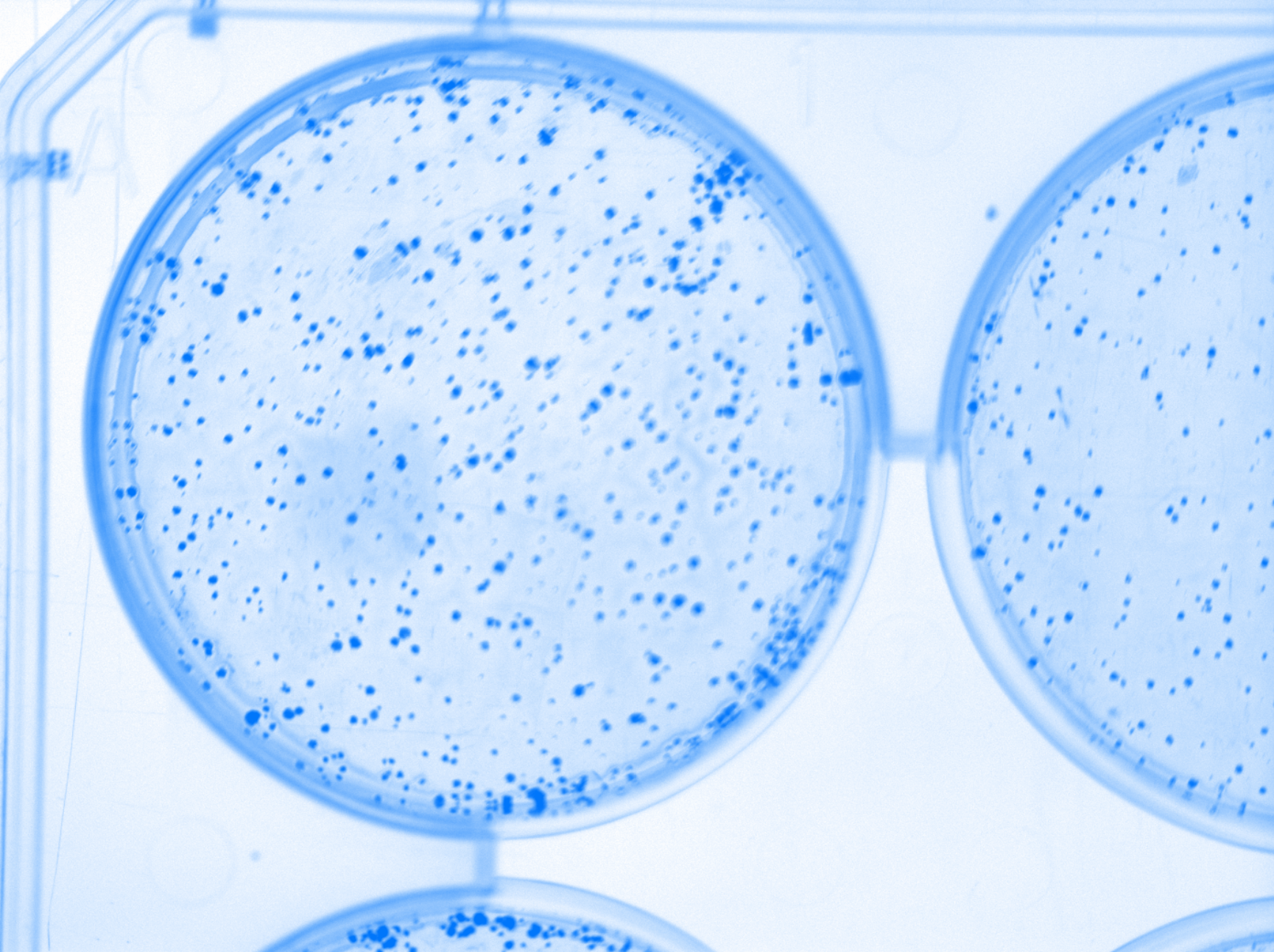

Supplement: Supplementary file 11 — Figure EV5 Source Data [file 44319_2025_576_MOESM11_ESM.zip › Figure EV5/EV5B/Control shRNA.tif]

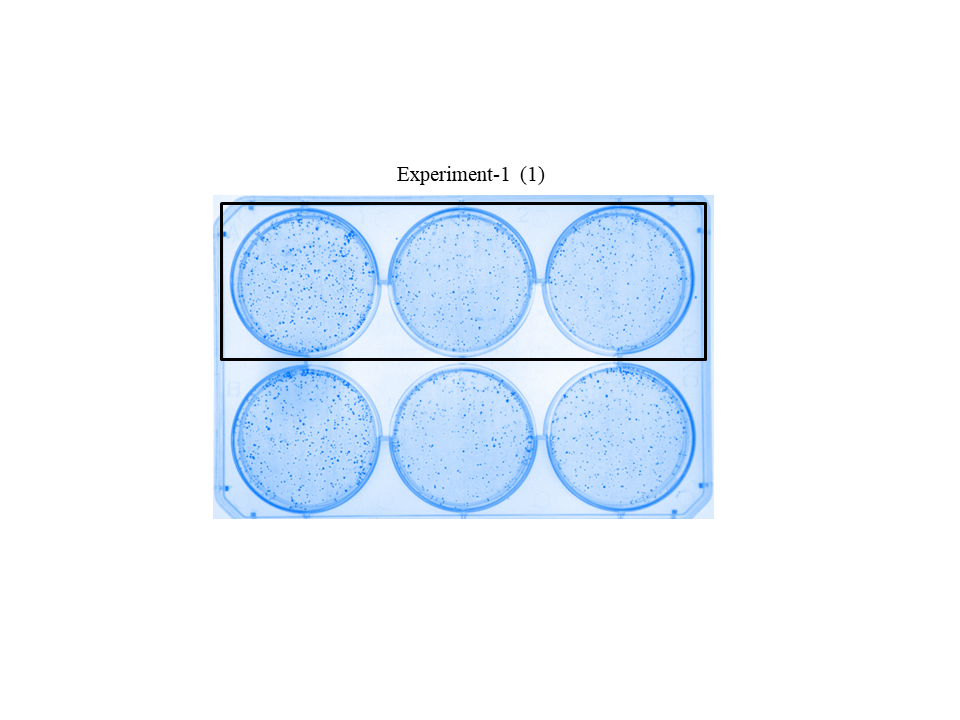

Supplement: Supplementary file 11 — Figure EV5 Source Data [file 44319_2025_576_MOESM11_ESM.zip › Figure EV5/EV5B/Full Plate_All Samples Together.tif]

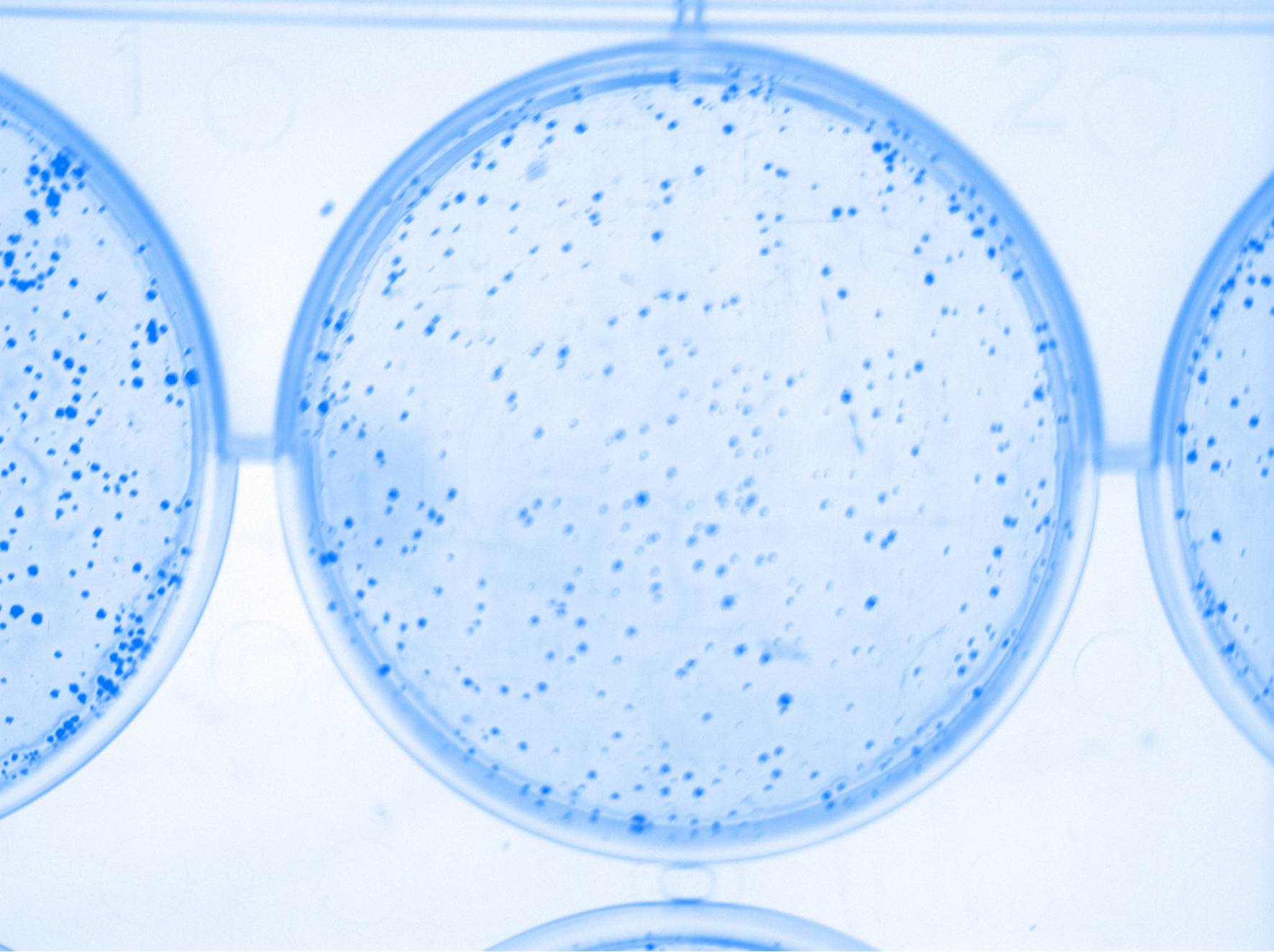

Supplement: Supplementary file 11 — Figure EV5 Source Data [file 44319_2025_576_MOESM11_ESM.zip › Figure EV5/EV5B/PTPN22 shRNA-1.tif]

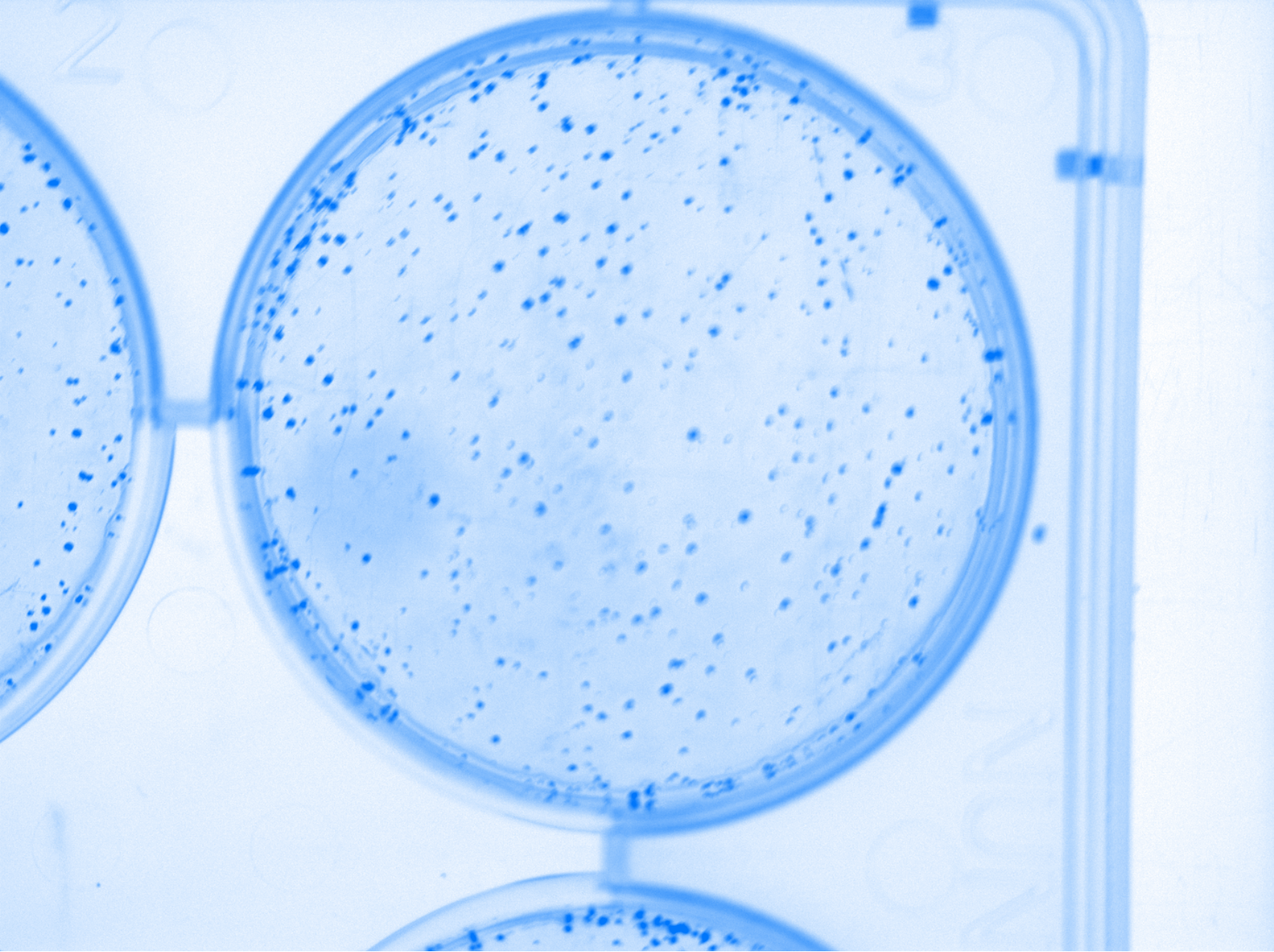

Supplement: Supplementary file 11 — Figure EV5 Source Data [file 44319_2025_576_MOESM11_ESM.zip › Figure EV5/EV5B/PTPN22 shRNA-2.tif]

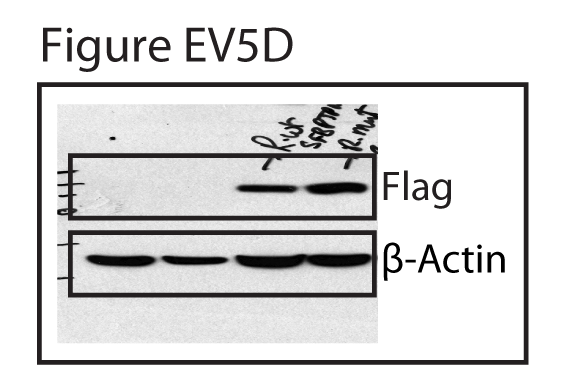

Supplement: Supplementary file 11 — Figure EV5 Source Data [file 44319_2025_576_MOESM11_ESM.zip › Figure EV5/EV5D/EV5D.tif]

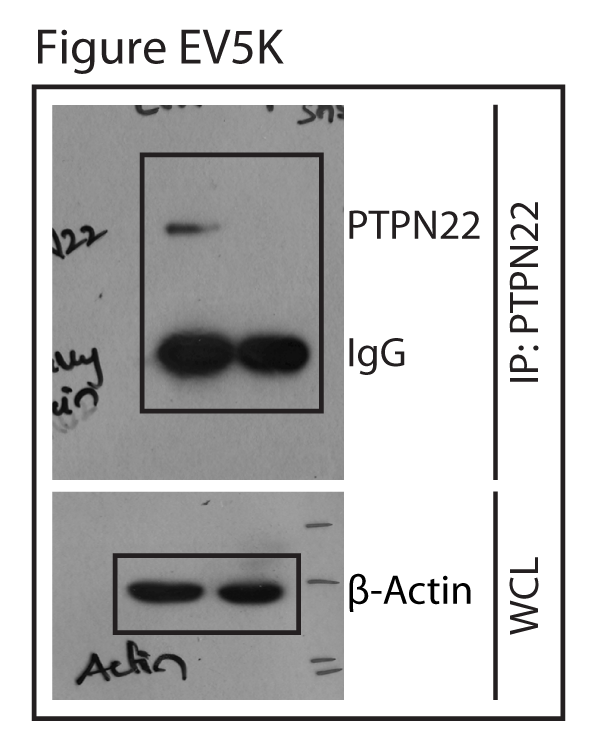

Supplement: Supplementary file 11 — Figure EV5 Source Data [file 44319_2025_576_MOESM11_ESM.zip › Figure EV5/EV5K/EV5K.tif]
