# Supplementary material for: Phosphatase PTPN22 functions as an adaptor in the mTORC2 complex
Source: EMBO Rep. 2025 Sep 16;26(21):5172–98. doi: 10.1038/s44319-025-00576-5 (PMC12592532; doi:10.1038/s44319-025-00576-5)
Supplement: Supplementary file 12 — Expanded View Figures [file 44319_2025_576_MOESM12_ESM.pdf]

# Expanded View Figures

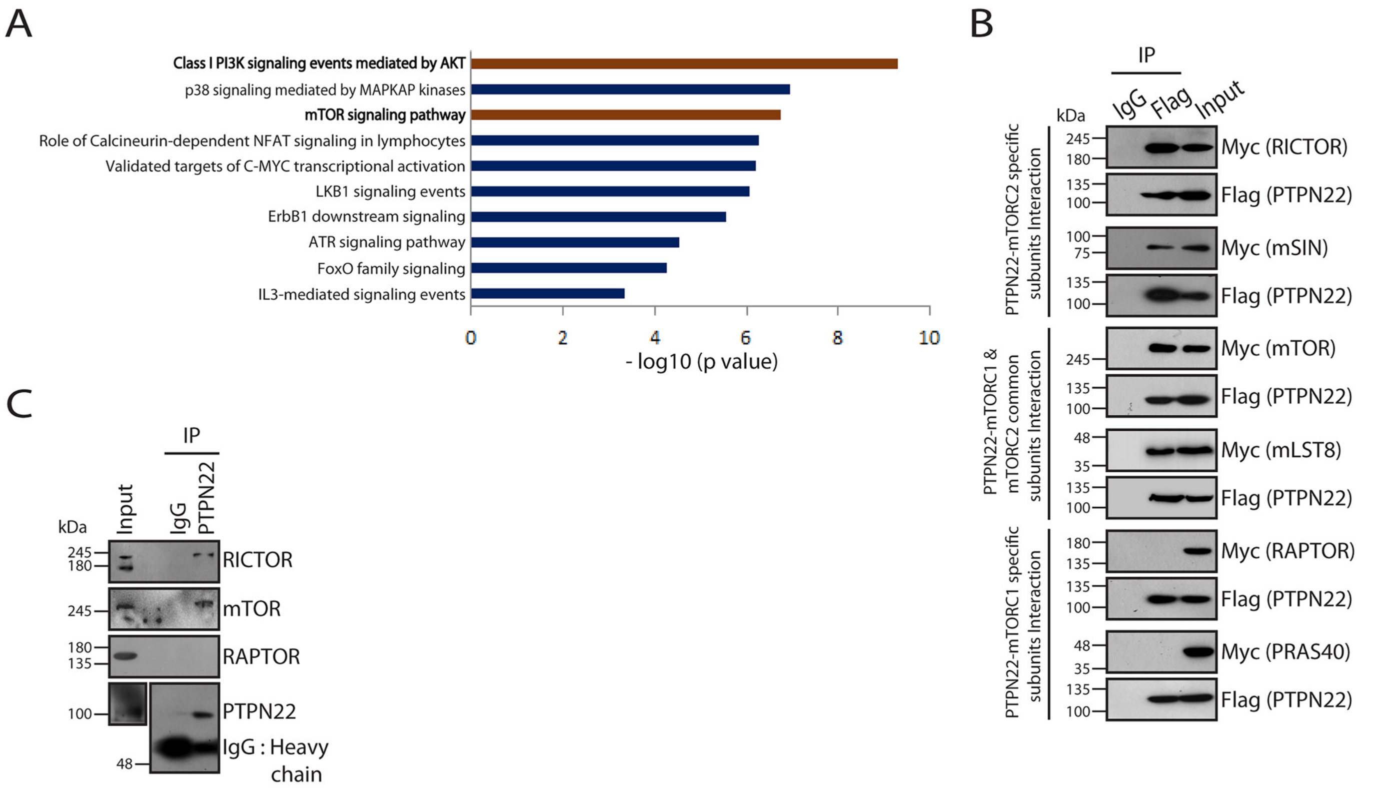


**Figure EV1. PTPN22 specifically binds to mTORC2 components, related to Fig. 1.**

(**A**) Bar graphs showing the −log10 *P* values of the most enriched pathways in the interactome of PTPN22 derived from NCI-pathways (Nature 2016) using Enrichr. (**B**) HEK293T cells were co-transfected with SFB-PTPN22 along with Myc-tagged RICTOR, mSIN, mTOR, mLST8, RAPTOR and PRAS40. 24 h after transfection, cells were lysed in 0.3% CHAPS buffer and the lysates were immunoprecipitated with control IgG or anti-Flag antibody. The interactions were detected by immunoblotting with anti-Myc antibody. (**C**) Immunoprecipitation (IP) with control IgG or anti-PTPN22 antibody was performed with extracts derived from HeLa cells. Endogenous association of PTPN22 with mTOR complexes subunits (RICTOR, mTOR, and RAPTOR) were analysed by immunoblotting with specific antibodies. Due to low expression level of PTPN22 in HeLa cells, endogenous PTPN22 in input sample was shown by immunoprecipitating PTPN22 from cell extracts using its antibody. Source data are available online for this figure.

 © The Author(s)

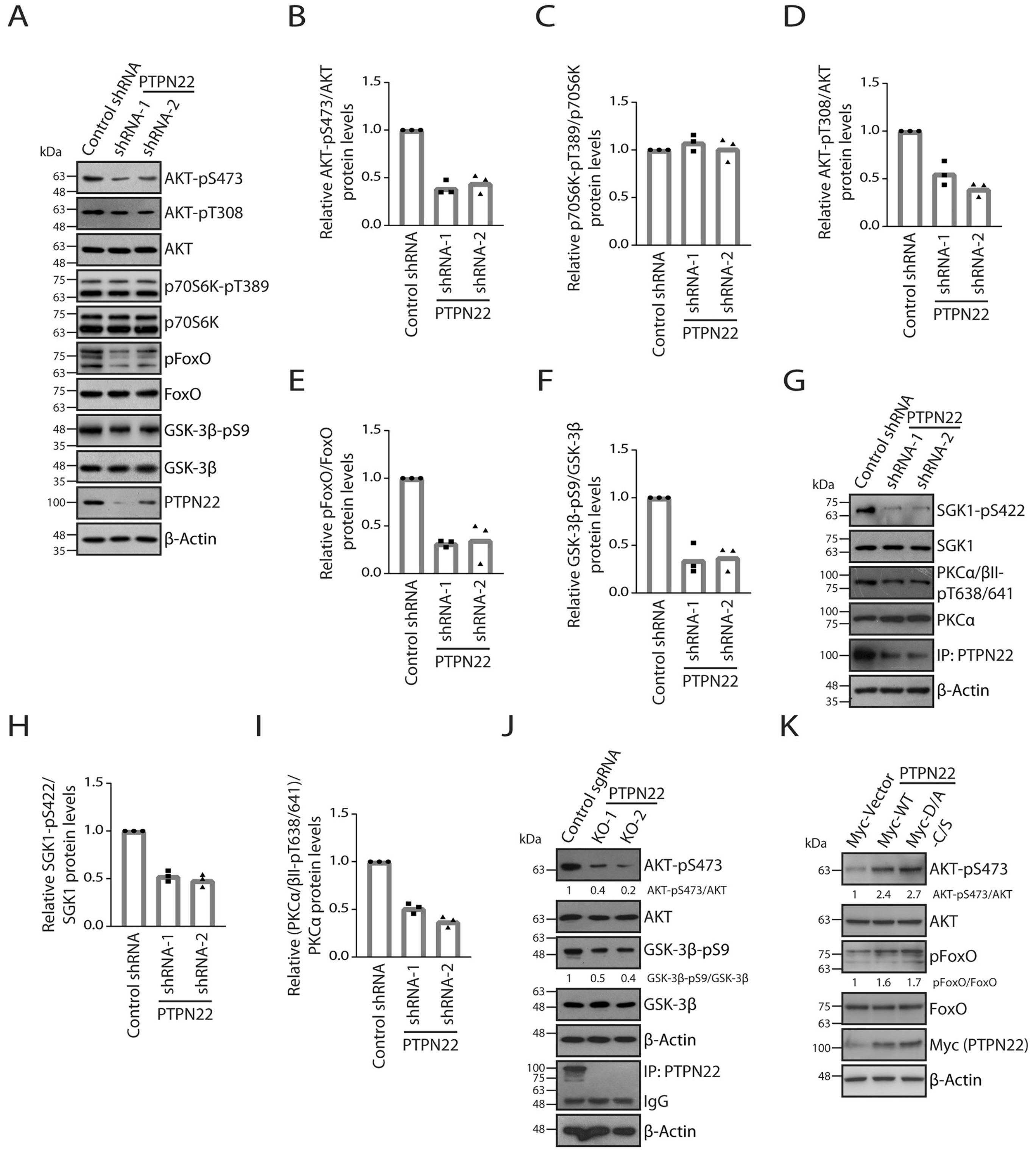
A
Control shRNA
PTPN22
shRNA-1
shRNA-2
kDa
AKT-pS473
AKT-pT308
AKT
p70S6K-pT389
p70S6K
pFoxO
FoxO
GSK-3β-pS9
GSK-3β
PTPN22
β-Actin
B
Relative AKT-pS473/AKT protein levels
C
Relative p70S6K-pT389/p70S6K protein levels
D
Relative AKT-pT308/AKT protein levels
E
Relative pFoxO/FoxO protein levels
F
Relative GSK-3β-pS9/GSK-3β protein levels
G
SGK1-pS422
SGK1
PKCα/βII-pT638/641
PKCα
IP: PTPN22
β-Actin
H
Relative SGK1-pS422/SGK1 protein levels
I
Relative (PKCα/βII-pT638/641)/PKCα protein levels
J
Control sgRNA
PTPN22
KO-1
KO-2
AKT-pS473
1 0.4 0.2 AKT-pS473/AKT
AKT
GSK-3β-pS9
1 0.5 0.4 GSK-3β-pS9/GSK-3β
GSK-3β
β-Actin
IP: PTPN22
IgG
β-Actin
K
Myc-Vector
PTPN22
Myc-WT
Myc-D/A -C/S
AKT-pS473
1 2.4 2.7 AKT-pS473/AKT
AKT
pFoxO
1 1.6 1.7 pFoxO/FoxO
FoxO
Myc (PTPN22)
β-Actin

**Figure EV2. PTPN22 knockdown inhibits mTORC2 activation, related to Fig. 2.**

(**A**) Representative immunoblots showing analysis of whole cell lysates derived from Jurkat cells depleted of endogenous PTPN22 by two independent shRNAs (shRNA-Scramble used as a negative control) via lentiviral mediated infection, for examine the total and phosphorylated states of AKT, p70S6K, GSK-3β and FoxO with their respective antibodies. (**B–F**) Quantification of immunoblotting data from (**A**). The pixel intensity of phosphorylated protein bands were normalized by the pixel intensity of corresponding total protein bands. Individual data points from three independent experiments are shown in the graph. (**G–I**) Representative immunoblots showing analysis of whole cell lysates derived from HCT116 cells depleted of endogenous PTPN22 by two independent shRNAs (shRNA-Scramble used as a negative control) via lentiviral mediated infection, for examine the total and phosphorylated states of SGK1 and PKCα with their respective antibodies (**G**). Quantification of SGK1 phosphorylation (**H**) and PKCα phosphorylation (**I**) from (**G**). Individual data points from three independent experiments are shown in the graph. (**J**) Cell extracts of control and PTPN22 knockout cells (derived from two independent guide RNAs) were analysed by immunoblotting to determine the total and phosphorylated states of AKT and GSK-3β with specific antibodies. (**K**) Immunoblots showing analysis of whole cell lysates derived from Jurkat cells transfected with Myc vector, Myc-tagged PTPN22 (WT) or PTPN22 (D/A-C/S) constructs, for determine the total and phosphorylated states of AKT and FoxO with specific antibodies. Source data are available online for this figure.

 © The Author(s)

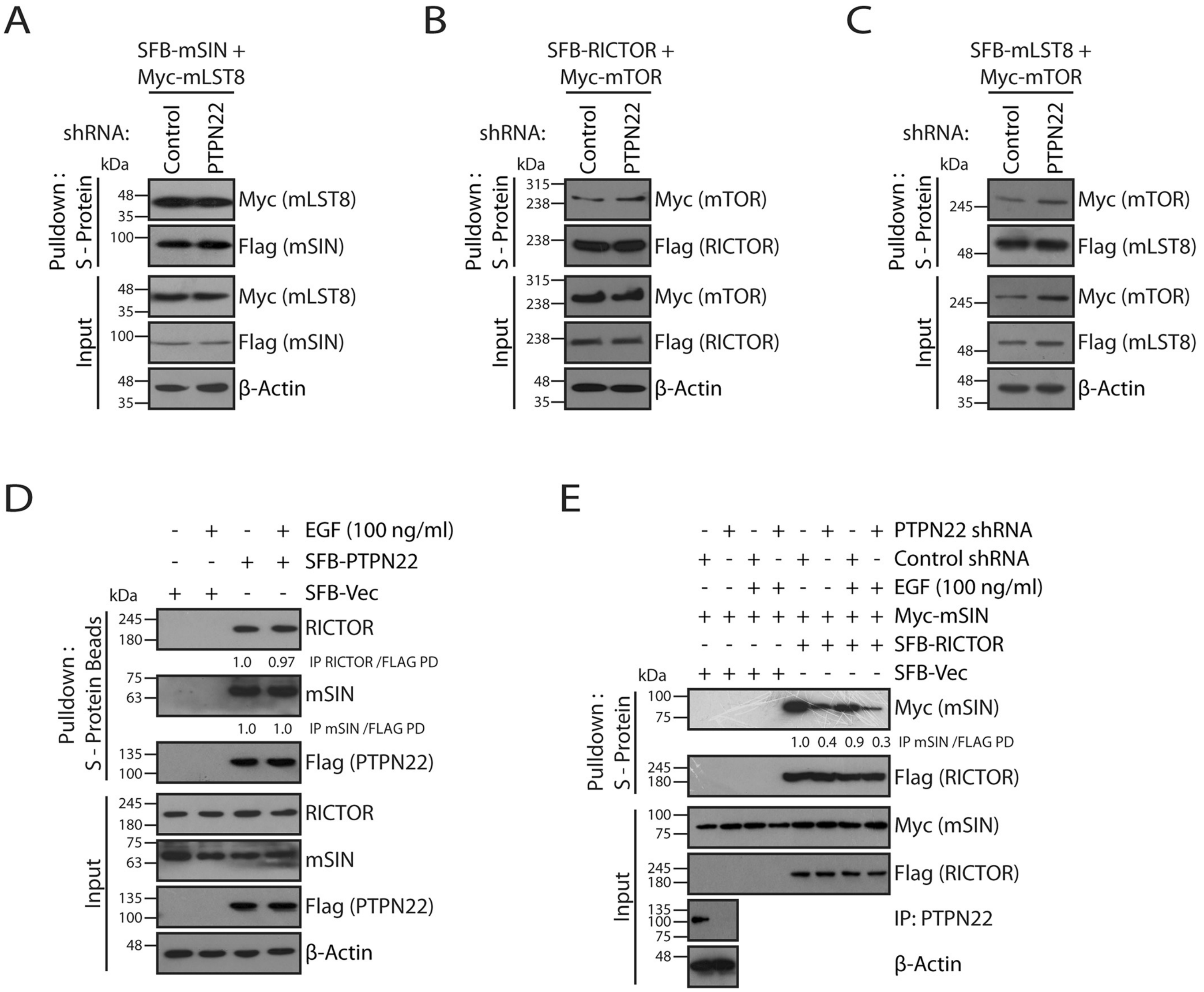


**Figure EV3. PTPN22 regulates mSIN and RICTOR association independent of growth factors, related to Fig. 3.**

(**A–C**) Control or PTPN22 depleted HCT116 cells were co-transfected either with (**A**) SFB-mSIN and Myc-mLST8, (**B**) SFB-RICTOR and Myc-mTOR, or (**C**) SFB-mLST8 and Myc-mTOR. At 48 h post-transfection, cells were lysed in 0.3% CHAPS buffer and lysates were pulldown using S-protein agarose beads. The interactions were detected by immunoblotting with anti-Myc antibody. (**D**) HEK293T cells were transfected with either SFB vector or SFB-PTPN22. At 24 h post-transfection, cells were serum starved for 16 h, followed by stimulation with EGF (100 ng/ml) for 15 min and lysed in 0.3% CHAPS buffer and were subjected to pulldown using S-protein agarose beads. Association of PTPN22 with RICTOR and mSIN were detected by immunoblotting with their respective antibodies. (**E**) Control or PTPN22 depleted HCT116 cells were co-transfected either with SFB vector or SFB-RICTOR along with Myc-mSIN. At 48 h post-transfection, cells were serum starved for 16 h, followed by stimulation with EGF (100 ng/ml) for 15 min. Cells were lysed in 1% Triton X-100 buffer and lysates were pulldown using S-protein agarose beads. The interactions were detected by immunoblotting with anti-Myc antibody. Source data are available online for this figure.

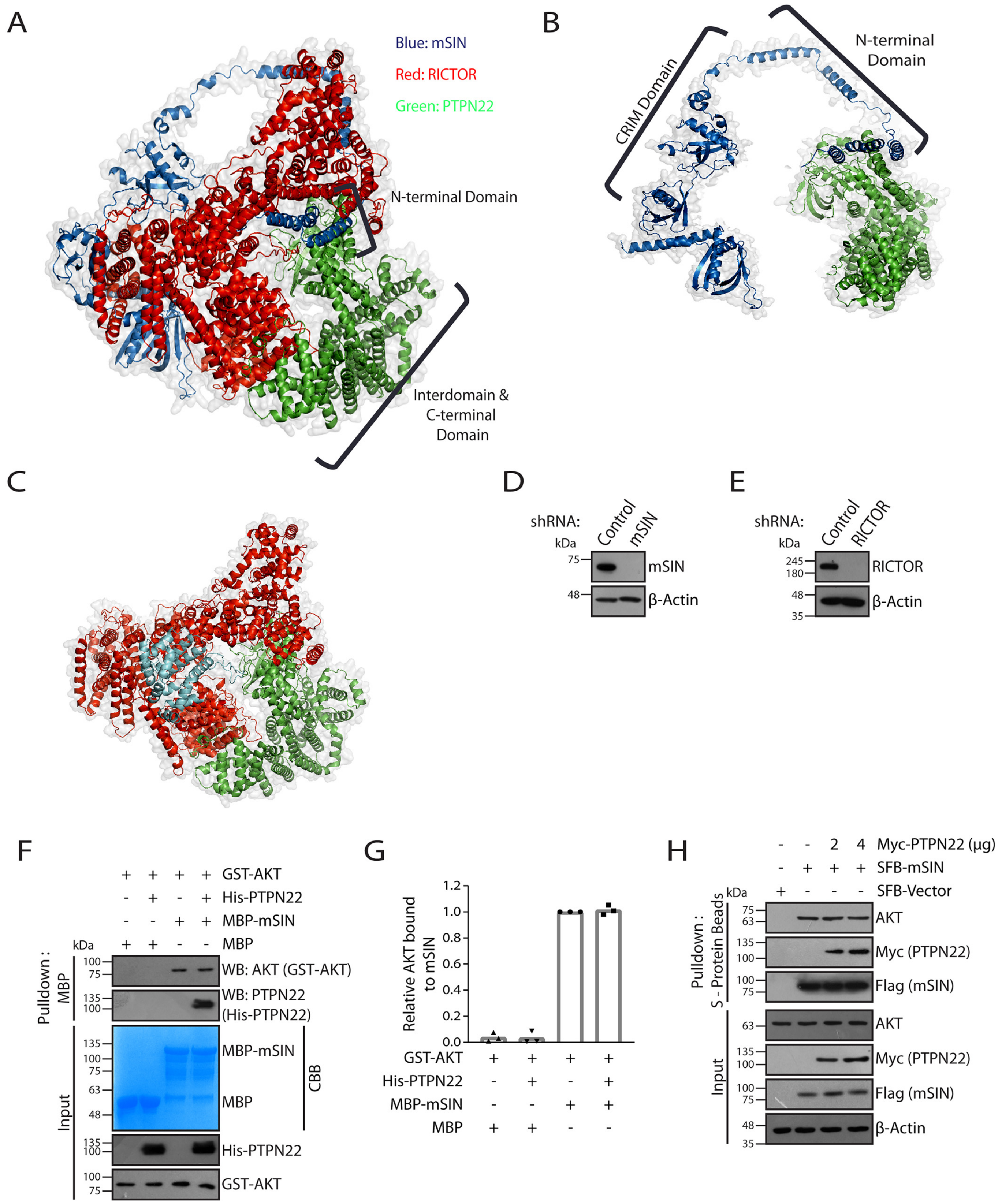
A
Blue: mSIN
Red: RICTOR
Green: PTPN22
N-terminal Domain
Interdomain & C-terminal Domain
B
CRIM Domain
N-terminal Domain
C
D
shRNA: Control mSIN
kDa
75
48
mSIN
β-Actin
E
shRNA: Control RICTOR
kDa
245
180
48
35
RICTOR
β-Actin
F
+ + + + GST-AKT
- + - + His-PTPN22
- - + + MBP-mSIN
+ + - - MBP
kDa
Pulldown : MBP
WB: AKT (GST-AKT)
WB: PTPN22 (His-PTPN22)
Input
MBP-mSIN
MBP
CBB
His-PTPN22
GST-AKT
G
Relative AKT bound to mSIN
1.2
1.0
0.8
0.6
0.4
0.2
0.0
GST-AKT + + + +
His-PTPN22 - + - +
MBP-mSIN - - + +
MBP + + - -
H
- - 2 4 Myc-PTPN22 (μg)
- + + + SFB-mSIN
+ - - - SFB-Vector
kDa
Pulldown : S - Protein Beads
AKT
Myc (PTPN22)
Flag (mSIN)
Input
AKT
Myc (PTPN22)
Flag (mSIN)
β-Actin

 © The Author(s)

**Figure EV4. Characterization of RICTOR-mSIN-PTPN22 interacting regions, related to Fig. 4.**

(**A–C**) The predicted Alphafold model of RICTOR-mSIN-PTPN22 complex is shown and the proteins are highlighted in indicated colours: mSIN (*blue*), RICTOR (*red*), and PTPN22 (*green*). The N-terminus and Interdomian along with C-terminal domain of PTPN22 are denoted by the labels. ipTM = 0.49 and pTM = 0.48 represents the confidence score of the predicted structure (**A**). (**B**) 3D model depicting PTPN22 and mSIN interaction, from the predicted structure. N-terminus and the CRIM domain of mSIN are denoted by labels. (**C**) 3D model depicting PTPN22 and RICTOR interaction, from the predicted structure. The *cyan* colour indicates the ARM4 region of RICTOR. (**D**) HEK293T cells were transduced with either control shRNA or pool of multiple mSIN shRNAs, via lentiviral mediated infection. Stable cell lines were generated and knockdown efficiency was verified by immunoblotting with anti-mSIN antibody. (**E**) HEK293T cells were transduced with either control shRNA or pool of multiple RICTOR shRNAs, via lentiviral mediated infection, and stable cell lines were generated. The knockdown efficiency was verified by immunoblotting with anti-RICTOR antibody. (**F, G**) MBP or MBP-mSIN bound on dextran sepharose beads were incubated with purified GST-AKT either in the presence of recombinant PTPN22 or equal volume of corresponding buffer, and its effect on the AKT-mSIN interaction was assessed by immunoblotting MBP-pulldowns with AKT antibody (**F**), and individuals data points for relative AKT bound to mSIN were plotted from three independent experiments (**G**). (**H**) SFB-mSIN along with increasing concentrations (0–4 µg plasmid) of Myc-PTPN22 were transfected in HEK293T cells. At 24 h post-transfection, cells were lysed and subjected to pulldown using S-protein agarose beads, and the effect of PTPN22 on the interaction of AKT and mSIN was assessed by immunoblotting pulldowns with AKT specific antibody. SFB vector was used as a negative control. β-actin was used as a loading control. Source data are available online for this figure.

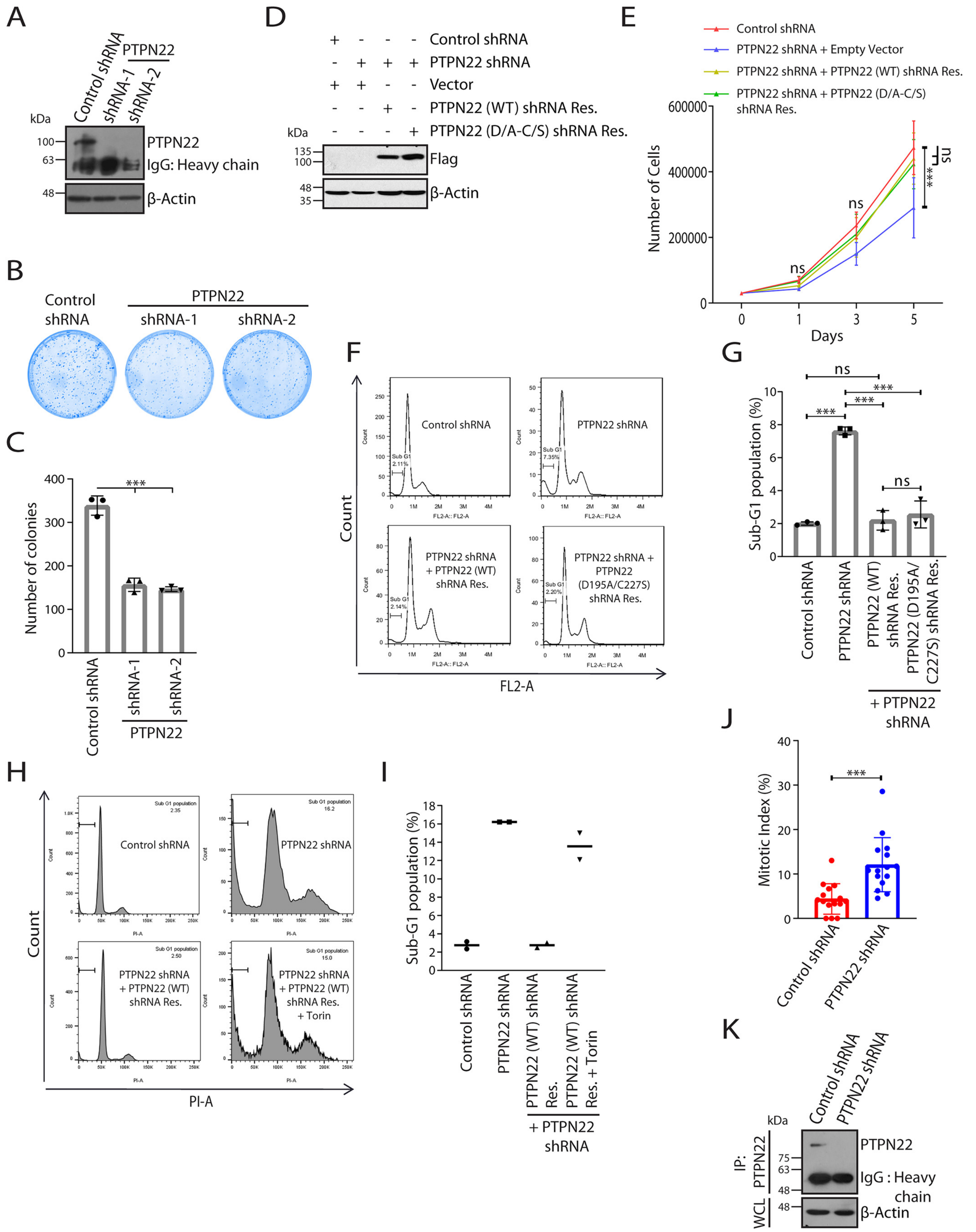
A
Control shRNA
PTPN22
shRNA-1
shRNA-2
kDa
100
63
48
PTPN22
IgG: Heavy chain
β-Actin
D
Control shRNA
PTPN22 shRNA
Vector
PTPN22 (WT) shRNA Res.
PTPN22 (D/A-C/S) shRNA Res.
kDa
135
100
48
35
Flag
β-Actin
E
Control shRNA
PTPN22 shRNA + Empty Vector
PTPN22 shRNA + PTPN22 (WT) shRNA Res.
PTPN22 shRNA + PTPN22 (D/A-C/S) shRNA Res.
Number of Cells
Days
ns
***
B
Control shRNA
PTPN22
shRNA-1
shRNA-2
C
Number of colonies
***
Control shRNA
shRNA-1
shRNA-2
PTPN22
F
Count
Control shRNA
PTPN22 shRNA
PTPN22 shRNA + PTPN22 (WT) shRNA Res.
PTPN22 shRNA + PTPN22 (D195A/C227S) shRNA Res.
FL2-A
G
Sub-G1 population (%)
ns
***
Control shRNA
PTPN22 shRNA
PTPN22 (WT) shRNA Res.
PTPN22 (D195A/C227S) shRNA Res.
+ PTPN22 shRNA
H
Count
Control shRNA
PTPN22 shRNA
PTPN22 shRNA + PTPN22 (WT) shRNA Res.
PTPN22 shRNA + PTPN22 (WT) shRNA Res. + Torin
PI-A
I
Sub-G1 population (%)
Control shRNA
PTPN22 shRNA
PTPN22 (WT) shRNA Res.
PTPN22 (WT) shRNA Res. + Torin
+ PTPN22 shRNA
J
Mitotic Index (%)
***
Control shRNA
PTPN22 shRNA
K
Control shRNA
PTPN22 shRNA
kDa
75
63
48
IP: PTPN22
WCL
PTPN22
IgG : Heavy chain
β-Actin

 © The Author(s)

**Figure EV5. PTPN22 acts as a potential oncogene, related to Fig. 5.**

(**A**) HCT116 cells were transduced with either control shRNA or two independent PTPN22 shRNAs via lentiviral mediated infection, and stable cell lines were generated. Knockdown efficiency was shown by immunoprecipitating PTPN22 from cell lysates using PTPN22 antibody, followed by immunoblot analysis of anti-PTPN22 immunoprecipitates and whole cell lysates with specific antibodies. (**B**, **C**) Colony formation assays were performed in HCT116 cells expressing control shRNA or PTPN22 shRNA constructs. (**B**) Images were captured after staining with crystal violet, and (**C**) quantification data for number of colonies from three independent experiments are shown. Error bars indicate mean ± standard deviation ($n = 3$), ***$P = 0.000024$ for control shRNA v/s PTPN22 shRNA-1, ***$P = 0.000018$ for control shRNA v/s PTPN22 shRNA-2 (one-way ANOVA with Bonferroni's multiple comparisons test). (**D**) The expression of shRNA-resistant PTPN22 wild-type (WT) or PTPN22 catalytic mutant (D/A-C/S) constructs transfected in PTPN22 depleted cells was shown by immunoblotting with anti-Flag antibody. (**E**) HCT116 cells expressing either control shRNA or PTPN22 shRNAs were transfected with indicated constructs. After day 1, cell number was measured every 2 days for the indicated durations. Quantification of cell number was calculated from three independent experiments. Error bars indicate mean ± SD ($n = 3$), ***$P = 0.0003$, ns: not significant (two-way ANOVA with Tukey's multiple comparisons test). (**F**, **G**) Representative images from sub-G1 population measurement by flow cytometric analysis in HCT116 cells expressing indicated constructs are shown (**F**), and (**G**) quantified data for percentage of sub-G1 population from three independent experiments were plotted. Error bars represent mean ± standard deviation ($n = 3$), ***$P = 0.00000616$ for control shRNA v/s PTPN22 shRNA, ***$P = 0.00000803$ for PTPN22 shRNA v/s PTPN22 shRNA + PTPN22 (WT) shRNA Res., ***$P = 0.0000135$ for PTPN22 shRNA v/s PTPN22 shRNA + PTPN22 (D195A/C227S) shRNA Res., ns: not significant for control shRNA v/s PTPN22 shRNA + PTPN22 (WT) shRNA Res., and for PTPN22 shRNA + PTPN22 (WT) shRNA Res. v/s PTPN22 shRNA + PTPN22 (D195A/C227S) shRNA Res. (one-way ANOVA with Bonferroni's multiple comparisons test). (**H**, **I**) Representative images from sub-G1 population measurement by flow cytometric analysis in HCT116 cells expressing indicated constructs, and were treated either with torin (250 nM) or with DMSO control for 2 h are shown (**H**), and scatter plot for percentage of sub-G1 population from two independent experiments were plotted (**I**). (**J**) Quantification data for the percentage of mitotic index (number of pH3 positive cells/total number of cells (DAPI) per field) in PTPN22 depleted cells as compared to the control cells were plotted. The plotted data points were from three biological replicates and represent mean ± SD, ***$P = 0.0002$ (unpaired two-tailed Student's *t* test). (**K**) Immunoblot showing PTPN22 knockdown efficiency in cell line used for nude mice xenograft experiments. Source data are available online for this figure.
